# OCT-based optic neuropathy diagnosis using explainable and privacy-preserving machine learning

**DOI:** 10.1038/s41598-026-53687-x

**Published:** 2026-05-26

**Authors:** Md Mahmudul Hasan, Jack Phu, Henrietta Wang, Michael Kalloniatis, Erik Meijering, Arcot Sowmya

**Affiliations:** 1 School of Computer Science and Engineering, University of New South Wales, Sydney, NSW 2052 Australia; 2 School of Optometry and Vision Science, University of New South Wales, Sydney, NSW 2052 Australia; 3 Centre for Eye Health, University of New South Wales, Sydney, NSW 2052 Australia; 4 Faculty of Medicine and Health, University of Sydney, Camperdown, NSW Australia; 5 School of Medicine (Optometry), Deakin University, Waurn Ponds, VIC Australia; 6 University of Houston College of Optometry, University of Houston, Houston, TX USA

**Keywords:** Optical coherence tomography, Glaucoma, Ischaemic optic neuropathy, Dementia, Parkinson’s disease, Perimetry, Explainable machine learning, SHAP analysis, Partial dependency analysis, Computational biology and bioinformatics, Diseases, Medical research, Neurology, Neuroscience

## Abstract

Glaucoma shares similarities with neurodegenerative conditions like dementia, Parkinson’s disease, and ischaemic optic neuropathy, which affect ocular health. However, current studies exclude neurodegenerative cases in neuropathy diagnosis and rely on ‘black box’ models. To address this, we applied explainable machine learning to optical coherence tomography (OCT) data, integrating a privacy-preserving mechanism to create a reliable neuropathy diagnostic tool. OCT data from 268 glaucomatous, 334 normal, 56 dementia, 60 Parkinson’s, and 93 ION eyes were analysed from a Sydney-based eye clinic. Spatial and frequency domain features were extracted, followed by feature selection and hierarchical classification. Model interpretability was enhanced using SHapley Additive exPlanations and partial dependency analysis, and privacy was preserved incorporating a differential privacy mechanism. A team of three clinicians, with 12, 11, and 6 years of experience, evaluated their performance on the same dataset for a human versus machine comparison, with the machine achieving an area under the curve of 0.90 for classifying neuropathy. Overall, machine outperformed clinicians, with 26.3% higher accuracy for neuropathy and 24.8% higher accuracy for glaucoma diagnosis. In conclusion, both non-explainable and explainable methods show promise in enhancing diagnostic support for clinical decision-making, with the privacy-preserving approach safeguarding data privacy.

**Supplementary Information:**

The online version contains supplementary material available at 10.1038/s41598-026-53687-x.

## Introduction

Glaucoma is a neurodegenerative disease primarily affecting the eye, and shares a common thread with other neurodegenerative conditions, including dementia, Parkinson’s disease, and ischaemic optic neuropathy (ION), all of which have secondary implications for ocular health^1^. According to ongoing research in this area, patients with glaucoma exhibit a significantly higher risk of developing secondary neurodegenerative diseases, such as dementia^2^ than individuals without glaucoma (hazard ratio [HR] = 1.13, 95% confidence interval [CI]: 1.01–1.27)^3^. Generally, both glaucoma and dementia are neurodegenerative disorders that share similarities in terms of neuronal loss, which leads to visual dysfunction and cognitive impairment, respectively. Several pieces of evidence link these two conditions, such as structural indicators, particularly degenerative changes observed in the ganglion cells^4,5^. While both diseases become more common with advancing age, this factor alone may not fully explain the higher co-occurrence of these conditions observed in various studies^4^.

Similar to dementia, Parkinson’s disease is a progressive neurodegenerative disorder of the central nervous system that affects movement and is also responsible for reduction in retinal nerve fibre layer (RNFL) and ganglion cell (GC) thickness^6^. ION, a condition that occurs due to the lack of blood flow to the optic nerve, also results in the death of retinal ganglion cells and their axons^7^. Given the confounding effects of neurodegenerative diseases, glaucoma can be challenging to detect, and most of the population can remain underdiagnosed. In contrast, Founti et al.^8^ performed a cross-sectional study on a mainly Caucasian population (2,554 participants) and reported that 60% of the population was overdiagnosed with glaucoma. That means more than half of the population diagnosed with glaucoma may not have glaucoma.

A recent review^1^ has revealed that most of the AI-based methods applied to glaucoma diagnosis excluded other conditions such as neurodegenerative diseases and ischemic optic neuropathy and vice-versa^1^. However, these conditions can mimic glaucoma and thus cause misdiagnosis. Given the characteristic changes in RNFL and GC, it is challenging for health care practitioners to differentiate between the diseases. Therefore, it is crucial to perform differential diagnosis of the primary neurodegenerative conditions, such as glaucoma, and secondary conditions mimicking glaucoma, such as dementia and ischaemic optic neuropathy.

Based on the aforementioned research findings, there is a growing body of evidence suggesting that computer-aided techniques could enhance glaucoma and cognitive neurodegeneration diagnosis more cost-effectively^1^. As many AI-based studies are based on publicly available fundus image datasets and trained black-box type end-to-end deep learning models^1^, there are several issues hindering real-life clinical applicability, such as explainability and privacy. A review of prior studies in this area shows that explanation of the identified features and the interpretation of machine learning models have received less focus than improvement of the performance metrics^1^, preventing stakeholders from understanding the reasons behind their decisions, and hindering the use of AI-based diagnostic solutions in the market^1^.

According to Raja and colleagues^9^, explainability and privacy are two essential parts of a trustworthy machine learning model. As such, it is important to unbox the black-box model for diagnostics to understand and explain how the tool makes its predictions, thereby building trust, reliability, transparency, and accountability, while also ensuring that its predictions are fair and unbiased in the critical decision-making process^10^. It is also important to ensure that the patients’ sensitive and critical information remain private in the decision-making process, which requires a robust privacy preserving mechanism. These two issues remain under-addressed when dealing with diagnosis of glaucoma and other neurodegenerative diseases using machine learning, which raises concerns about the trustworthiness of the system.

The core contribution of this study is the use of a unique patient cohort comprising patients with glaucoma, ION, dementia, and Parkinson’s disease to perform machine learning (ML) based differential diagnosis between these conditions—a novel approach not explored in previous studies. To the best of our knowledge, explainable analysis using the SHapley Additive exPlanations (SHAP) and partial dependency analysis (PDA) has not yet been performed in the context of optic neuropathy diagnosis, which also uses a privacy preserving scheme as a layer of security. The current study sought to assess the trustworthiness of an OCT-based optic neuropathy diagnostic tool, with a focus on explainability and protecting privacy, in order to build a trustworthy and user-friendly software tool to aid clinicians in diagnosing neuropathy.

## Materials and methods

### Data collection

Ethics approval was granted by the University of New South Wales (HC210563), and all participants gave written informed consent allowing their de-identified clinical data to be used in research. The study followed the principles of the Declaration of Helsinki. Data was collected from the patients who visited the Centre for Eye Health (CFEH) between 2015 and 2021. Diagnosis of glaucoma and other ocular diseases (including ION) was undertaken as per CFEH protocols^11–14^, which include review of the clinical data by a senior clinician senior optometrist or ophthalmologist. Additionally, a third expert conducted further examinations for the purpose of inclusion in the study. In summary, glaucoma assessment at the clinic involved a comprehensive evaluation comprising patient history, visual acuity testing, anterior segment examination, applanation tonometry, pachymetry, gonioscopy, and dilated stereoscopic examination of both the optic nerve head and macula. This was complemented by standard automated perimetry, colour fundus photography of the optic disc and posterior pole, and OCT imaging. Individuals identified as “glaucoma suspects” or those with non-glaucomatous optic atrophy were excluded from the study. Among the other neurodegenerative diseases, the dementia and Parkinson’s patients were identified by self-reports and medications, which were found in their medical history. When a neurodegenerative disease patient was concurrently found to be affected by glaucoma, they were excluded from the cohort. During the phenotyping process, clinicians flagged any images with artefacts (Supplementary Fig. 1(a, b)). It is worth noting that CFEH is a referred-only clinic and diagnosis and clinical audits also exist within the centre^14^.

All scans were obtained using the CIRRUS HD-OCT 500 (Carl Zeiss Meditec, Dublin, CA, USA), a spectral-domain OCT system that acquires high-resolution volumetric retinal and optic nerve head images, including fundus images and cross-sectional B-scans. The CIRRUS HD-OCT 500 uses a line-scanning ophthalmoscope (LSO) fundus imaging camera for alignment and scanning, along with a CCD iris camera (1280 × 1024 resolution) for patient positioning. The OCT system provides ~ 5 µm axial resolution, ~ 15 µm transverse resolution, and commonly acquires 512 × 128 cube scans (128 B-scan slices with 512 A-scans per slice) over a 6 mm × 6 mm retinal region. Scans were captured using the device’s standard high-density protocol with automated focus, centring, and optimisation, and only images with signal strength ≥ 6 were retained to ensure image quality.

#### Data labelling

A total of 334 healthy eyes were included from 167 patients (Supplementary Fig. 1(c)). The neuropathy cohort includes 268 glaucomatous optic neuropathy (ON) and 209 non-glaucomatous ON eyes (Table 1). The non-glaucomatous optic neuropathy includes ION and neurodegenerative disease patients. Among the neurodegenerative conditions, we included 28 dementia patients (n = 56 eyes), 30 Parkinson’s patients (n = 60 eyes) and 93 ION patients (n = 93 eyes). ION cases include those that are considered either secondary optic atrophy or consecutive optic atrophy. Secondary optic atrophy cases included conditions such as ION and optic neuritis, while consecutive optic atrophy cases included retinal vascular occlusive disease that caused RNFL loss. All the ION cases caused RNFL loss and associated optic atrophy. Advanced glaucoma patients identified based on mean deviation (MD) and central field defect (CFD) were included in glaucoma cohort. Example OCT images for each class are provided in Supplementary Fig. 1(d)).

**Table 1:** Demographics of subjects included in this study.

Glaucoma types	Number of patients (N)	Age (mean + /− std)	Number of right eye (RE)	Number of left eye (LE)	Number of RE + LE
Normal	167	67.77 ± 6.85	167	167	334
Early Glaucoma	86	66.34 ± 8.86	48	38	86
Moderate Glaucoma	72	65.90 ± 11.68	36	36	72
Advanced Glaucoma (based on MD)	37	64.70 ± 10.83	24	13	37
Advanced Glaucoma (based on CFD)	73	61.78 ± 13.39	39	34	73
Glaucomatous ON (Total)	268	64.48 ± 11.44	147	121	268
Dementia	28	74.19 ± 11.84	28	28	56
Parkinson’s	30	68.64 ± 9.67	30	30	60
Ischaemic Optic Neuropathy (ION)	93	59.20 ± 12.75	45	48	93
Non-glaucomatous ON (Total)	151	65.93 ± 13.27	103	106	209
Glaucomatous + Non-glaucomatous ON (Total)	419	65.09 ± 12.27	250	227	477

N: Number of subjects, LE: Left Eye, RE: Right eye, std: Standard deviation

The categorised data were used to design a hierarchical classification system with two levels of binary classifiers from the included cohort (Supplementary Fig. 1(c)). A hierarchical binary classification framework was selected over a direct multiclass approach to better reflect the clinical diagnostic workflow and to mitigate class imbalance between optic neuropathy subtypes. This approach also enables more sensitive detection of early pathology.

Top-level classifier (Classifier 1): This classifier distinguishes between Normal and ON cases, where ON includes glaucomatous ON, and non-glaucomatous ON (dementia, Parkinson’s disease and ION).

Sub-level classifier (Classifier 2): Within the ON category identified by the top-level classifier, this classifier differentiates between glaucoma and non-glaucoma conditions (which include dementia, Parkinson’s disease, and ION).

Additionally, three other one-versus-one (OvO) classifiers were trained and tested within this hierarchical framework to separately distinguish glaucoma from each of the neurodegenerative diseases: glaucoma versus dementia, glaucoma versus Parkinson’s disease, and glaucoma versus ION. A sub-analysis was conducted to differentiate between non-glaucomatous conditions using three additional OvO classifiers: ION versus dementia, ION versus Parkinson’s, and dementia versus Parkinson’s. Each of the classifiers were developed using three different machine learning algorithms. As we have two classifiers (Classifier 1: optic neuropathy vs normals, Classifier 2: glaucoma vs non-glaucoma), the advanced cases were always included in the glaucoma cohort, and no separate classifier was used for classification between advanced glaucoma identified based on MD vs CFD.

### Feature extraction in spatial and frequency domain

**Fig. 1:**
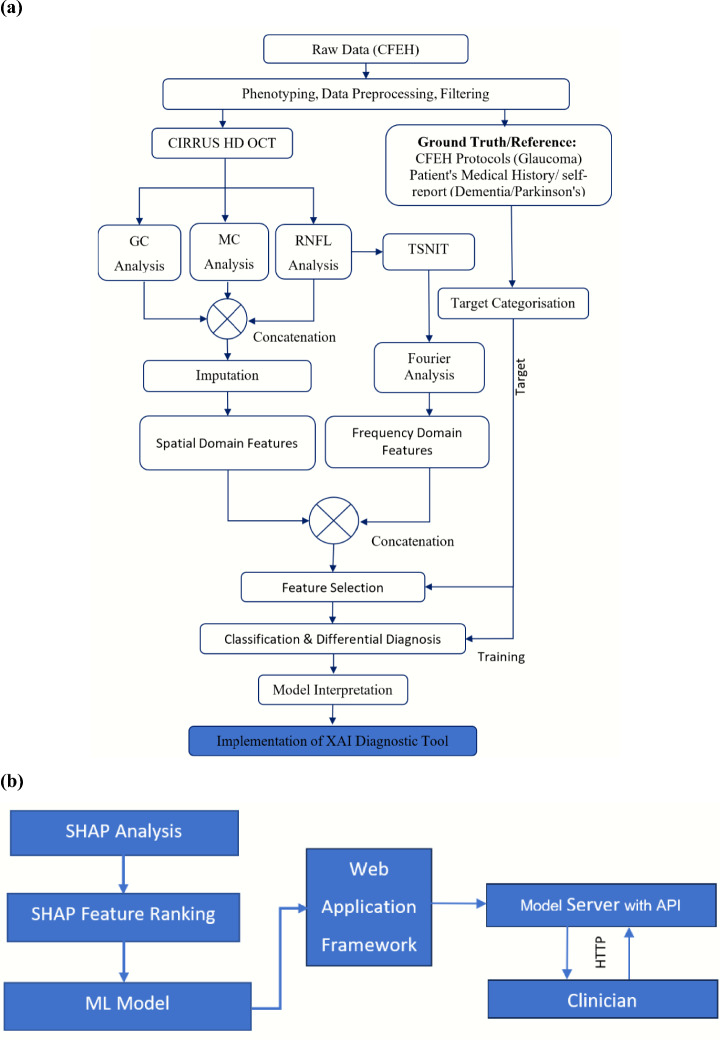
(**a**) Flow chart of the experimental study. The study involves raw data phenotyping, spatial and frequency domain feature extraction, feature selection, classification, model interpretation and app development using explainable machine learning (XML). (**b**) Schematic of the implementation process of the diagnostic tool based on XML (non-private). Blue shaded blocks represent explainable analysis.

Spatial domain features from 2D OCT images were extracted using the CIRRUS HD-OCT software from CFEH^15^ (Fig. 1a, Supplementary Fig. 2, Supplementary Table 1). Statistical computation was performed on the 256 data points of the temporal-superior-nasal-inferior-temporal (TSNIT) patterns extracted from RNFL analysis, which is a comprehensive profile of the RNFL thickness around the optic disc, measured in microns. The pattern was further used to generate some statistical features (e.g., skewness, kurtosis) and information theoretic features (e.g., Shannon entropy, Fisher information). A Fast Fourier transformation (FFT)^16^ was applied to the ‘double hump TSNIT pattern’ in the spatial domain to generate several frequency-domain features (Supplementary Table 1).

#### Feature imputation and selection

In certain cases, patient data exhibited artefacts, including image truncation, media opacities, and segmentation errors, impacting measurements of RNFL/GC/Macular (MC) thickness. We included patients with at least one artefact-free measurement (e.g., RNFL), while measurements with artefacts (e.g., GC) were treated as missing data and were generated with multiple imputation using chained equations (MICE)^17^. Optimising classification model performance requires the elimination of irrelevant features^18^. To this end, we utilised filter methods for their independence from specific learning algorithms and proven effectiveness across diverse datasets^19–21^. We first employed Pearson’s correlation coefficient to identify features that are highly correlated (r > 0.98). Then the ANOVA F Test was run on the same feature sets, and finally, the most significant features determined by the F-scores were included, excluding those with lower rank and higher correlation with a top-ranked feature in the spatial-frequency domain. The remaining features were ranked by their F-scores, and the ‘SelectKBest’ scikit-learn module^22^ was applied, selecting the K highest-scored features with the wrapper approach^23^. In this approach, a tuned classification model with five-fold cross-validation is used to determine the optimal cut-off by observing validation accuracy with an increasing number of top-ranking features from the spatial-frequency domains. It is important to note that for feature importance, clean data was used, and therefore MICE imputation did not affect the feature importance.

### Classification

Currently, a standardised model for neuropathy classification does not exist. Considering the unique computational demands and complexities involved^24,25^, and drawing insights from the literature, we adopted k-nearest neighbour (KNN), support vector machine (SVM), and random forests (RF) classification algorithms, which are three distinct supervised classification algorithms to train the models. Through an iterative process, we fine-tuned the hyperparameters of each classifier to achieve optimal accuracy post cross-validation, employing a grid-search algorithm for RF and SVM (Supplementary Table 2). For KNN, we selected the 'k' value associated with the best training accuracy, adjusting the k-value within the range: 1 ≤ k ≤ 40 (Supplementary Fig. 3). Prior to inputting features into the ML models, feature scaling was executed, involving min–max scaling and standardisation respectively for KNN and SVM^26^. RF did not undergo feature scaling, given its intrinsic algorithmic characteristics, contributing to enhanced feature interpretation through explainable machine learning (XML). To address data imbalance, we incorporated a synthetic minority over-sampling technique (SMOTE)^27^. All models were trained using Python version 3.7 on Google Colab^28^.

#### Performance evaluation, patient-level splitting and cross-validation

Machine learning classifier performance was assessed using sensitivity, specificity, AUC, F1-score, and accuracy scores. To mitigate underfitting arising from imbalanced data, the receiver operating characteristic curve (ROC) was plotted, an optimal point identified in the plot using geometric mean and the metrics were reported for this optimal point. As the dataset includes both left and right eyes as separate samples for the same patients in certain cases, training on one eye and testing on the other may lead to data leakage^29,30^. To address this, patient-level splitting was applied, where each patient’s pair of eyes was assigned to either the training or test dataset. Additionally, five-fold cross-validation^31^ was employed across all classification models to assess their robustness, with mean and standard deviations reported for evaluation.

### Performance evaluation by clinicians

Once the performance of the machine learning models was evaluated, the same data was provided to three experienced clinicians to grade the images on classifications: first, neurodegenerative versus normal, and if neurodegenerative, glaucoma versus non-glaucoma. The clinicians are highly trained and experienced (12, 11 and 6 years) at CFEH—a referral-only optometry-ophthalmology service. Clinicians were provided with masked deviation maps only and did not have access to additional clinical metadata such as age, medical history, or longitudinal progression data. This was done to standardise comparison with the model.

### Explainable machine learning

#### Shapley additive analysis

SHapley Additive exPlanations (SHAP) is a method for interpreting machine learning predictions by attributing the predictions to influential features, utilising Shapley values from game theory^32^. These values determine the importance of each feature in influencing a model’s prediction, and have been applied in various domains, including medical^33^ and other contexts^34,35^. The Shapley value for a feature *i*, denoted as ${\varphi}_{i}\left(\mathrm{v}\mathrm{a}\mathrm{l}\right)$[36], is computed as:

1 $$\varphi _{i} \left( {{\mathrm{val}}} \right) = ~\mathop \sum \limits_{{S \subseteq \left[ {1, \ldots ,m} \right]\backslash \left[ i \right]}} \frac{{\left\lfloor S \right\rfloor !\left( {m - \left\lfloor S \right\rfloor - 1} \right)!}}{{m!}}\left( {{\mathrm{val}}\left( {S \cup \left[ i \right]} \right) - {\mathrm{val}}\left( S \right)} \right)$$

where *i, m,* and *S* represent the feature under consideration, total number of features excluding *i*, and the subset of all features excluding *i*, respectively. The term $\mathrm{v}\mathrm{a}\mathrm{l}\left(S\right)$ represents the predicted value (e.g., output of the model) when the model is only considering the subset *S* of features. $\mathrm{v}\mathrm{a}\mathrm{l}\left(S\cup [i]\right)$ represents the predicted value when the model considers the subset *S* of features plus the additional feature *i*. It shows the model’s output when feature *i* is included along with all the features in *S*. The difference value $(\mathrm{v}\mathrm{a}\mathrm{l}\left(S\cup [i]\right)-\mathrm{v}\mathrm{a}\mathrm{l}\left(S\right))$ represents the marginal contribution of feature *i* when added to the subset *S*. The SHAP framework, extending and generalising Shapley values, is commonly referred to as ‘SHAP values’^36^.

#### Partial dependency analysis

Partial dependency analysis (PDA)^37^ is a method used to interpret ML models, specifically exploring the relationship between an individual feature and the model’s output, while keeping all other features unchanged. PDA has found extensive application in real-world problem interpretation^34,35^, particularly for discerning decision boundaries^38–40^ in classification problems using ML. PDA quantifies this relationship as follows:

2 $${\widehat{f}}_{i}(i)= {E}_{S}\left[{\widehat{f}}_{i}\left(i, S\right)\right]= \int {\widehat{f}}_{i}\left(i, S\right)d{\mathbb{P}}(S)$$

where *i* represents the feature of interest, ${\widehat{f}}_{i}\left(i, S\right)$ denotes the predicted outcome for feature *i* based on the ML model, and $d{\mathbb{P}}(S)$ represents the probability distribution. The function is approximated using the Monte Carlo method [36]:

3 $${\widehat{f}}_{i}\left(i\right)= \frac{1}{n}\sum_{j=1}^{n}{\widehat{f}}_{i}\left(i, {S}^{(j)}\right)$$

### XML-based diagnostic tool

Utilising the SHAP-based assessment of feature importance, we have created a web-based application featuring a deployed RF model. To deploy the ML model, at first the model was integrated into a web application framework (Fig. 1(b)), and then it was deployed to a server, where clinicians can input the necessary features and obtain the predicted output from the ML model as a neuropathy likelihood, and if it is neuropathy, then the likelihood of glaucoma and non-glaucoma. This tool utilises the key features identified via SHAP analysis, allowing the model to decide the feature importance blended on the RF model. To validate the likelihood scores generated by the application, predicted probabilities from the web app were plotted against the MD values obtained from visual fields data for each sample.

### Privacy preserving mechanism

#### Differential privacy

Differential privacy (DP) is a method for safeguarding privacy that employs random noise to ensure that when a single individual’s data in a dataset is modified, the publicly accessible information does not undergo substantial changes^41^. By preventing any single data point from strongly influencing the output, it makes it challenging for attackers to accurately deduce private information (such as patient’s age) associated with any particular data point^41^.

In our DP algorithm, dataset ${D}_{OCT}$ is the collection of all the OCT features with sensitive clinical information (e.g., age and gender). The query *Q* could be used for individual predictions, such as "predict the likelihood of neuropathy for a specific patient", since it takes OCT dataset ${D}_{OCT}$ as input and predicts a probability as output. The Laplacian mechanism ensures ε-differential privacy by adding Laplacian noise L to $Q \left({D}_{OCT}\right)$, resulting in an encrypted dataset $Q\left({{D}_{OCT}}{\prime}\right)$^42^:

4 $$Q\left({{D}_{OCT}}{\prime}\right)=Q \left({D}_{OCT}\right)+ \mathrm{L}$$

The Laplacian noise level is controlled by a privacy budget epsilon (ε), with smaller values providing stronger privacy but potentially impacting the model’s performance^43^. The (ε, *σ*)-differential privacy condition *þorn* for the OCT dataset ($${D}_{OCT}$$ ), with a subset $ of possible output probabilities while maintaining privacy violation parameter σ^42^, is as follows:

5 $$\thorn\left[\mathrm{R}\left({D}_{OCT}\right)\in {\$}\right]\le \mathrm{e}\mathrm{x}\mathrm{p}\left(\upvarepsilon \right) \thorn\left[\mathrm{R}\left({{D}_{OCT}}{\prime}\right)\in {\$}\right]+ \upsigma$$

Here, R represents a randomised algorithm or mechanism that processes the OCT dataset ${D}_{OCT}$ to ensure differential privacy. By applying Ɍ, the mechanism introduces controlled noise to dataset, ensuring that the privacy of individuals within the dataset is protected according to the *\upvarepsilon* -differential privacy standard. Noise is applied post-training at the prediction stage. Differential privacy noise is applied during model inference at the prediction stage, after the model has been fully trained. While the trained model does not store identifiable patient data, differential privacy protects against potential inference attacks by preventing reconstruction of training samples from prediction outputs.

#### Deployment of XML-based private model

We implemented the ML model with a privacy-preserving (PP) mechanism, most specifically differential privacy. To do this, we trained a RF model with differential privacy enabled by tuning and setting the epsilon parameter. The latter represents the privacy budget, which determines the amount of noise added to the model to protect privacy. A higher epsilon allows for more accurate predictions but provides less privacy and vice-versa. To deploy the XML model, we created a Flask web application for users to input data and receive predictions (Supplementary Fig. 4). The Flask app uses ‘ngrok’ for tunneling when it is run locally. When users input data into the web app, the privacy-preserving model is used to make predictions. The model adds differential privacy noise to the predictions to protect user privacy.

Once the XML model is deployed to a server, clinicians can input the necessary features and obtain the predicted output by the ML model as glaucoma likelihood. When the clinician uses the web application to input data, which might include sensitive information such as medical test results and the patient’s age, the entered data is transmitted to a server where the ML model is hosted. This transmission is done securely using encryption to protect data in transit. On the server, the data is processed by a privacy-preserving ML model, which not only protects privacy from external users like clinicians but also guards against privacy breaches by server personnel.

## Results

### TSNIT plots and statistical analysis

We generated a TSNIT plot displaying the spatial domain TSNIT pattern separately for normal, dementia, and glaucoma for both right (Supplementary Fig. 5(a)) and left (Supplementary Fig. 5(b)) eyes. The plots revealed a notable reduction (p < 0.05) in RNFL thickness values in the inferior and superior quadrants for left and right eyes, both for glaucoma and dementia patients. However, the reduction for the dementia group is substantially lower than for glaucoma, and very specific to the inferior and supertemporal region for the right eye, and superotemporal and inferotemporal region for the left eye. The difference plots of the TSNIT curves (Supplementary Fig. 5(c,d)), considering the normal value as the base, show the differences in different locations corresponding to the optic nerve head (ONH) but also the differences in the pattern of left and right eyes, especially in inferior region.

### Selected features

**Fig. 2:**
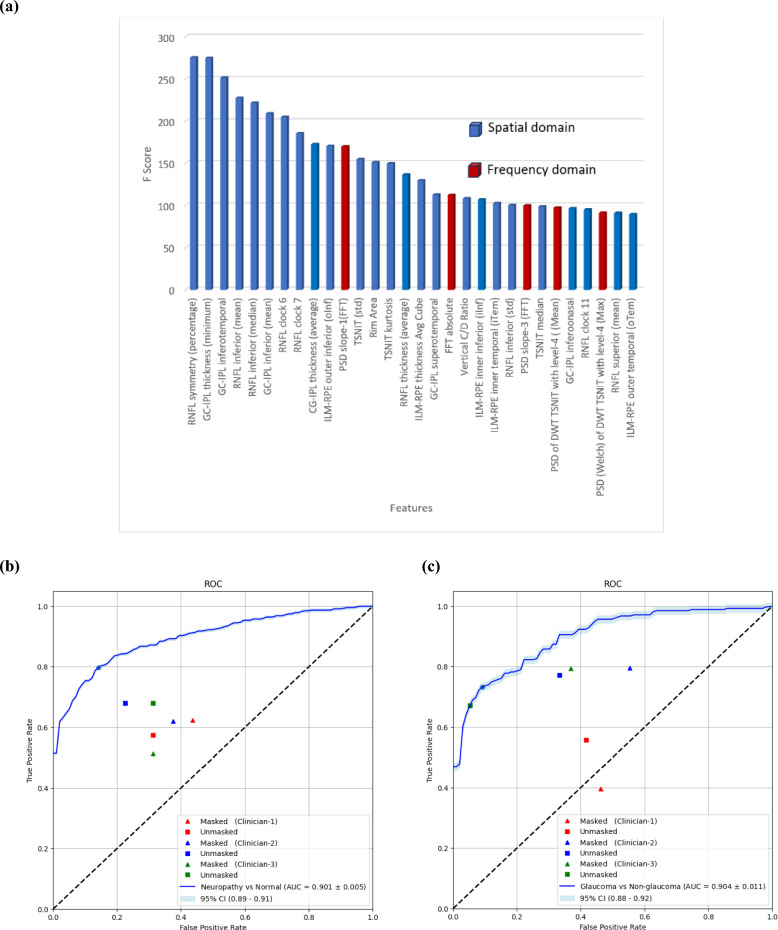
(**a**) Top 30 features selected by feature selection techniques. (**b**) Differential diagnosis of optic neuropathy versus normal (mean area under the ROC curve is 0.90 with fivefold cross validation using an RF classifier) and (**c**) Differential diagnosis of glaucomatous versus non-glaucomatous optic neuropathy (mean area under the ROC curve is 0.90 with fivefold cross validation using an RF classifier).

We conducted feature selection on a total of 131 spatial-frequency domain features obtained using the analysis with CIRRUS OCT software (Supplementary Table 1). Additionally, two clinical features (the patient’s age and gender) were included, which makes a total of 133 features. After eliminating strongly associated features (r > 0.98) (keeping the topmost with the highest F-score), the K highest ranked features were selected to observe the validation accuracy wrapping with an RF classifier. Adding features with F-scores below 10 did not contribute to improved validation accuracy; consequently, a cut-off F-score of 10 was established, corresponding to approximately 3.6% of the highest-ranked feature’s F-score (275.5). This resulted in the selection of 82 features (51 spatial domain features including one clinical feature and 31 frequency domain features) out of the initial 133 features (Supplementary Table 3 and Fig. 2a. The feature ranking highlighted RNFL and GC-IPL thickness features, specifically RNFL symmetry and GC-IPL thickness (minimum), as top-ranking features with highest score. Within frequency-domain features, the first slope of Power Spectral Density (PSD) (using FFT method) emerged as the highest-ranked feature.

### Differential diagnosis

#### Classifier 1: optic neuropathy versus normal

The differential diagnosis of optic neuropathy was assessed by several performance measures. While higher sensitivity and lower specificity may lead to increased false positives and overdiagnosis, the AUC provides a balanced evaluation of diagnostic performance by aggregating sensitivity and specificity under different classification thresholds. Therefore, our comparative analysis focuses on AUC. All classifiers demonstrated promising performance in diagnosing optic neuropathy (AUC range: 0.87–0.90), with KNN achieving the lowest AUC of 0.87 (95% CI 0.85–0.89), SVM recording an AUC of 0.89 (95% CI 0.87–0.93), and the RF-based model yielding the highest AUC of 0.90 (95% CI 0.89–0.91) (Table 2 and Supplementary Fig. 6).

**Table 2:** Performance of machine (three different classification models) and human experts in diagnosing primary and secondary neurodegeneration with fivefold cross validation. Best mean performance per metric is indicated in bold. Input features included RNFL, GC-IPL and macular thickness in spatial and frequency domain. 95% CI = Mean ± 1.96 × standard error (SE), SE = std / /$\sqrt{\mathrm{n}}$, n = 5, CI = confidence interval. Boldfaced values indicate the best performance among the classifiers/ human experts.

Machine/human		Optic Neuropathy Versus Normal					Glaucoma Versus Non-glaucoma				
Model	Folds	Sensitivity	Specificity	Accuracy	AUC	F1-Score	Sensitivity	Specificity	**Accuracy**	**AUC**	**F1-Score**
KNN	Fold-1	0.677	0.914	0.796	0.866	0.811	0.855	0.805	0.830	0.894	0.857
	Fold-2	0.724	0.828	0.776	0.856	0.814	0.942	0.795	0.869	0.896	0.891
	Fold-3	0.805	0.838	0.821	0.910	0.835	0.836	0.875	0.856	0.925	0.868
	Fold-4	0.735	0.833	0.784	0.857	0.821	0.796	0.878	0.837	0.893	0.852
	Fold-5	0.725	0.900	0.813	0.867	0.834	0.769	0.860	0.815	0.876	0.816
	**Mean ± SD**	0.734 ± 0.046	0.863 ± 0.041	0.798 ± 0.019	0.871 ± 0.022	0.823 ± 0.011	0.84 ± 0.066	0.843 ± 0.04	0.841 ± 0.021	0.897 ± 0.018	0.857 ± 0.027
	**95% CI**	0.694–0.774	0.827–0.899	0.781–0.815	0.852–0.890	0.813–0.833	0.782–0.898	0.808–0.878	0.823–0.859	0.881–0.913	0.833–0.881
SVM	Fold-1	0.734	0.863	0.798	0.871	0.823	0.926	0.857	0.892	0.953	0.909
	Fold-2	0.805	0.947	0.876	0.926	0.874	0.839	0.875	0.857	0.894	0.870
	Fold-3	0.742	0.957	0.849	0.880	0.857	0.820	0.844	0.832	0.904	0.843
	Fold-4	0.760	0.984	0.872	0.931	0.888	0.887	0.905	0.896	0.942	0.904
	Fold-5	0.800	0.968	0.884	0.901	0.879	0.964	0.825	0.894	0.943	0.923
	**Mean ± SD**	0.768 ± 0.033	**0.956 ± 0.023**	**0.862 ± 0.023**	0.899 ± 0.032	0.865 ± 0.024	**0.887 ± 0.059**	0.861 ± 0.03	**0.874 ± 0.028**	**0.927 ± 0.026**	**0.890 ± 0.033**
	**95% CI**	0.739–0.797	0.936–0.976	0.842–0.882	0.871–0.927	0.844–0.886	0.835–0.939	0.835–0.887	0.849–0.899	0.904–0.950	0.861–0.919
RF	Fold-1	0.785	0.857	0.821	0.888	0.845	0.855	0.878	0.866	0.900	0.879
	Fold-2	0.827	0.891	0.859	0.894	0.871	0.923	0.818	0.871	0.939	0.893
	Fold-3	0.829	0.925	0.877	0.909	0.872	0.782	0.925	0.853	0.914	0.851
	Fold-4	0.843	0.850	0.847	0.900	0.878	0.685	0.902	0.794	0.879	0.843
	Fold-5	0.853	0.850	0.851	0.916	0.891	0.731	0.907	0.819	0.887	0.850
	**Mean ± SD**	**0.827 ± 0.026**	0.875 ± 0.033	0.851 ± 0.02	**0.901 ± 0.011**	**0.871 ± 0.017**	0.795 ± 0.095	**0.886 ± 0.042**	0.841 ± 0.033	0.904 ± 0.024	0.863 ± 0.021
	**95% CI**	0.804–0.850	0.846–0.904	0.833–0.869	0.891–0.911	0.856–0.886	0.712–0.878	0.849–0.923	0.812–0.870	0.883–0.924	0.845–0.881
Human Experts	Clinician 1 (Masked)	**0.623**	0.563	0.604	–	0.674	0.397	0.538	0.436	–	0.505
	Clinician 2 (Masked)	0.620	0.625	**0.621**	–	**0.681**	**0.796**	0.448	0.688	–	0.778
	Clinician 3 (Masked)	0.513	**0.687**	0.573	–	0.611	0.793	**0.631**	**0.753**	–	**0.820**
	**Mean Clinicians’ Performance (Masked)**	0.585	0.625	0.599	–	0.655	0.662	0.539	0.626	–	0.701

#### Classifier 2: glaucoma versus non-glaucoma

A sub-classification was performed within optic neuropathy to observe how well the ML classifiers differentiate glaucoma cases from secondary conditions (non-glaucomatous optic neuropathies and neurodegenerative diseases). This classification was conducted using two approaches: one versus all (OvA) (Table 2) and one versus one (OvO) (Table 3), employing the three supervised models. The feature sets used for this classification were the same as those utilised in the main classifiers, because the same set of features is required to be used in the same pipeline to perform main and sub-classification for a specific patient.

**Table 3:** Performance of the three classification models in identifying various stages of glaucoma using fivefold cross-validation. The reported numbers are the mean ± standard deviation across the 5 folds. Input features consist of RNFL, GC-IPL, and macular thickness. The incorporation of advanced glaucoma cases is determined solely by the mean deviation of visual fields. Boldfaced values indicate the highest AUC using the best classifier.

			Glaucoma vs non-glaucoma (one vs one classifiers)			Intra-non-glaucomatous diseases (one vs one classifiers)		
Classifier	Metric		Glaucoma vs Dementia	Glaucoma vs Parkinson’s	Glaucoma vs Ischaemic ON	Ischaemic ON vs Dementia	Ischaemic ON vs Parkinson’s	Dementia vs Parkison’s
KNN	Sensitivity		0.854 ± 0.105	0.806 ± 0.062	0.811 ± 0.135	0.679 ± 0.161	0.771 ± 0.100	0.706 ± 0.209
	Specificity		0.819 ± 0.064	0.944 ± 0.052	0.807 ± 0.084	0.752 ± 0.242	0.632 ± 0.176	0.649 ± 0.184
	Accuracy		0.837 ± 0.033	0.875 ± 0.033	0.809 ± 0.064	0.716 ± 0.105	0.702 ± 0.080	0.677 ± 0.031
	AUC		0.882 ± 0.043	0.921 ± 0.024	0.871 ± 0.074	0.721 ± 0.162	0.702 ± 0.080	0.670 ± 0.079
	F1-Score		0.931 ± 0.014	0.936 ± 0.022	0.905 ± 0.027	0.801 ± 0.109	0.784 ± 0.081	0.759 ± 0.062
SVM	Sensitivity		0.899 ± 0.085	0.89 ± 0.066	0.869 ± 0.081	0.723 ± 0.099	0.755 ± 0.093	0.662 ± 0.159
	Specificity		0.948 ± 0.047	0.938 ± 0.100	0.868 ± 0.105	0.850 ± 0.192	0.865 ± 0.051	0.537 ± 0.200
	Accuracy		0.924 ± 0.050	0.914 ± 0.043	0.868 ± 0.075	0.786 ± 0.048	0.810 ± 0.062	0.599 ± 0.136
	AUC		**0.952 ± 0.032**	**0.957 ± 0.021**	**0.911 ± 0.066**	0.798 ± 0.053	**0.828 ± 0.058**	0.557 ± 0.196
	F1-Score		0.962 ± 0.017	0.962 ± 0.011	0.927 ± 0.041	0.851 ± 0.018	0.851 ± 0.042	0.722 ± 0.064
RF	Sensitivity		0.873 ± 0.063	0.854 ± 0.043	0.859 ± 0.049	0.811 ± 0.122	0.754 ± 0.108	0.688 ± 0.161
	Specificity		0.894 ± 0.073	0.951 ± 0.071	0.820 ± 0.094	0.863 ± 0.172	0.76 ± 0.143	0.750 ± 0.195
	Accuracy		0.883 ± 0.046	0.902 ± 0.037	0.840 ± 0.044	0.837 ± 0.032	0.757 ± 0.100	0.719 ± 0.079
	AUC		0.917 ± 0.046	0.943 ± 0.031	0.890 ± 0.038	**0.861 ± 0.068**	0.755 ± 0.083	**0.708 ± 0.087**
	F1-Score		0.951 ± 0.018	0.950 ± 0.021	0.918 ± 0.015	0.886 ± 0.020	0.813 ± 0.099	0.768 ± 0.054
AVERAGE	Sensitivity		0.875 ± 0.084	0.850 ± 0.057	0.846 ± 0.088	0.738 ± 0.127	0.760 ± 0.100	0.685 ± 0.177
	Specificity		0.887 ± 0.061	0.945 ± 0.074	0.832 ± 0.094	0.822 ± 0.202	0.752 ± 0.123	0.645 ± 0.193
	Accuracy		0.881 ± 0.043	0.897 ± 0.038	0.839 ± 0.061	0.780 ± 0.062	0.756 ± 0.081	0.665 ± 0.082
	AUC		0.917 ± 0.040	0.940 ± 0.025	0.891 ± 0.059	0.793 ± 0.094	0.761 ± 0.074	0.644 ± 0.121
	F1-Score		0.948 ± 0.017	0.949 ± 0.018	0.917 ± 0.028	0.846 ± 0.049	0.816 ± 0.074	0.750 ± 0.060

In the OvA classification (Table 2, Supplementary Fig. 7(a–c)), the classifiers were employed to classify glaucoma (primary) versus all other secondary neurodegenerative conditions (dementia, Parkinson’s, and ION). All classifiers exhibited encouraging performance, with AUC values ranging from 0.89 to 0.93. Specifically, KNN attained the lowest AUC of 0.89 (95% CI 0.88–0.91), RF reached an AUC of 0.90 (95% CI 0.88–0.92), and the SVM-based model yielded the highest AUC of 0.93 (95% CI 0.90–0.95) (Table 2).

In the OvO classification (Table 3, Supplementary Fig. 8(a–c)), when differentiating each of the non-glaucomatous conditions (secondary conditions) from glaucomatous cases (primary), the SVM model achieved an AUC of 0.95 (95% CI 0.92–0.98), 0.96 (95% CI 0.94–0.98) and 0.91 (0.85–0.97) for classifying dementia, Parkinson’s and ION, respectively, against glaucoma. Similarly, the RF classifier produced an AUC of 0.92 (95% CI 0.88–0.96), 0.94 (95% CI 0.92–0.97) and 0.89 (95% CI 0.86–0.92), respectively, while classifying dementia, Parkinson’s disease, and ION against glaucoma patients. A slightly lower performance was achieved using KNN, with AUC of 0.88 (95% CI 0.84–0.92), 0.92 (95% CI 0.90–0.94) and 0.87 (95% CI 0.81–0.94), respectively, while classifying dementia, Parkinson’s disease, and ION against glaucoma patients. Overall, SVM achieved the best AUC for the OvO classifications.

#### Intra-Class classification of non-glaucomatous ON

A sub-analysis was performed on differentiating between nonglaucomatous ON and neurodegenerative conditions. With the three conditions being present in the group (dementia, Parkinson’s diseases, and ION), we developed three different classifiers: ION versus dementia, ION versus Parkinson’s, and dementia versus Parkinson’s. For classifying ION versus dementia, RF achieved the highest AUC of 0.86 (95% CI 0.80–0.92), while for classifying ION versus Parkinson’s, SVM achieved the highest AUC of 0.83 (95% CI 0.78–0.88), and for classifying dementia versus Parkinson’s, the RF classifier attained the highest AUC of 0.71 (95% CI 0.63–0.78) (Table 3 and Supplementary Fig. 9(a–c)).

#### Human Versus Machine Performance

A sub-analysis was conducted to compare clinician performance with machine performance (Table 2, Supplementary Table 4) in differential diagnosis of neuropathy from normal (Fig. 2b) and glaucoma from non-glaucoma cases (Fig. 2c), with sensitivity and specificity metrics plotted in an ROC curve. Clinicians evaluated an image dataset in two ways: using masked deviation maps and OCT maps displaying only thickness values (Supplementary Fig. 10(a)) and using unmasked data with random distribution (Supplementary Fig. 10(b)).

Results showed that, using masked data, clinicians achieved a sensitivity of 51.3–62.3% and specificity of 56.3–68.3% for neuropathy diagnosis. For correctly identified neuropathy cases, sensitivity was 39.7–79.6% and specificity was 44.8–63.1% for distinguishing glaucoma from non-glaucoma (Supplementary Table 4). Machine learning outperformed clinicians, with a 26.3% higher accuracy for neuropathy and 24.8% higher accuracy for glaucoma using masked data (comparing with Table 2 data). While clinicians performed similarly with masked and unmasked data for neuropathy, their performance was slightly lower with masked data for glaucoma. However, in both scenarios, their performance was significantly lower than the machine’s, particularly for neuropathy diagnosis. The clinicians who were all highly trained and experienced demonstrated lower accuracy compared to the machine’s performance.

### Explainable ML results

#### SHAP analysis

**Fig. 3:**
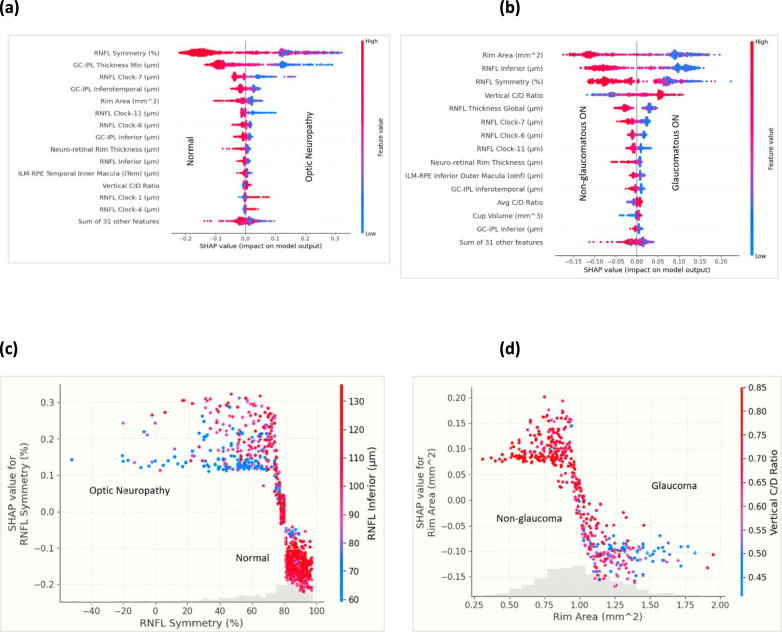
SHAP analysis findings: (**a**) SHAP-based feature ranking for Classifier 1. (**b**) SHAP-based feature ranking for Classifier 2. The summary plot using SHAP highlights the significance of these features, with blue indicating lower values and red indicating higher values. (**c**) SHAP dependence plot for Classifier 1 illustrates the relationship between RNFL symmetry and RNFL inferior thickness. The x-axis represents the RNFL Symmetry feature values, measured in percentage (%). The y-axis on the left side represents the corresponding SHAP values for RNFL Symmetry, and the legend on the right side shows the colour coding for RNFL Inferior thickness features, measured in micrometer (μm). The blue colour indicates the lower magnitude of RNFL Inferior thickness, while the red colour indicates the higher magnitude of the feature. (**d**) SHAP dependence plot for Classifier 2 illustrates the relationship between Rim area and vertical C/D ratio. The y-axis on the left side represents the corresponding SHAP values for Rim area, and the legend on the right side shows the colour coding for Vertical C/D Ratio features. The blue colour indicates the lower magnitude of Vertical C/D Ratio, while the red colour indicates the higher magnitude of the feature.

The global feature ranking and summary based on SHAP analysis for the RF model for both Classifier 1 (normal versus ON) (Fig. 3a) and Classifier 2 (glaucoma versus non-glaucoma) (Fig. 3b) were plotted for comparison. The SHAP feature ranking for Classifier 1 shows that RNFL symmetry is the topmost feature, followed by GC-IPL thickness value (minimum), RNFL clock-7 (part of RNFL inferior) and GC-IPL thickness at the inferotemporal area. On the other hand, for Classifier 2, the features change priority and Rim area appears to be the topmost ranked feature, pushing RNFL thickness at inferior quadrant to the second place. Other important features include RNFL symmetry and Vertical C/D ratio.

In the SHAP summary plots (Fig. 3), positive SHAP values observed on the right-hand side influence the model towards predicting a win, which in this context refers to a higher probability of classification as optic neuropathy (Fig. 3a) or glaucoma (Fig. 3b). Conversely, negative SHAP values displayed on the left side guide the model towards predicting a loss, indicating a higher likelihood of classification as normal (Fig. 3a) or non-glaucoma (Fig. 3b). These plots demonstrate that a decrease in RNFL symmetry (Fig. 3a) is correlated with an increased probability of being classified as optic neuropathy. Similarly, a decrease in Rim area (Fig. 3b) is associated with a heightened likelihood of being classified as glaucoma.

A dependence plot generated for key features (Fig. 3c, d) depicts the contribution of each feature’s magnitude to the model prediction and interaction with another feature at the same time. Positive SHAP values indicate a positive influence on prediction, negative values imply the opposite, and points near the zero-dashed line indicate minimal impact. The plot for Classifier 1 (Fig. 3c) shows that decreased RNFL symmetry heightens the probability of optic neuropathy classification, while increased RNFL symmetry tends towards normal classification, with a transition around 77 μm. Additionally, the interaction between RNFL symmetry and RNFL inferior thickness further amplifies the likelihood of optic neuropathy classification, offering additional clarity in interpretation that both being lower increases the chance of neuropathy. On the other hand, the SHAP dependence and interaction visualisation plot for Classifier 2 (Fig. 3d) shows that the lower the Rim area, the greater the probability of a glaucoma diagnosis, with a clear feature cut-off at 1 mm^2^, whereas the presence of higher C/D ratio with lower Rim area increases the chance and cross-validates the feature’s contribution to the machine learning model.

#### Partial dependency analysis

**Fig. 4:**
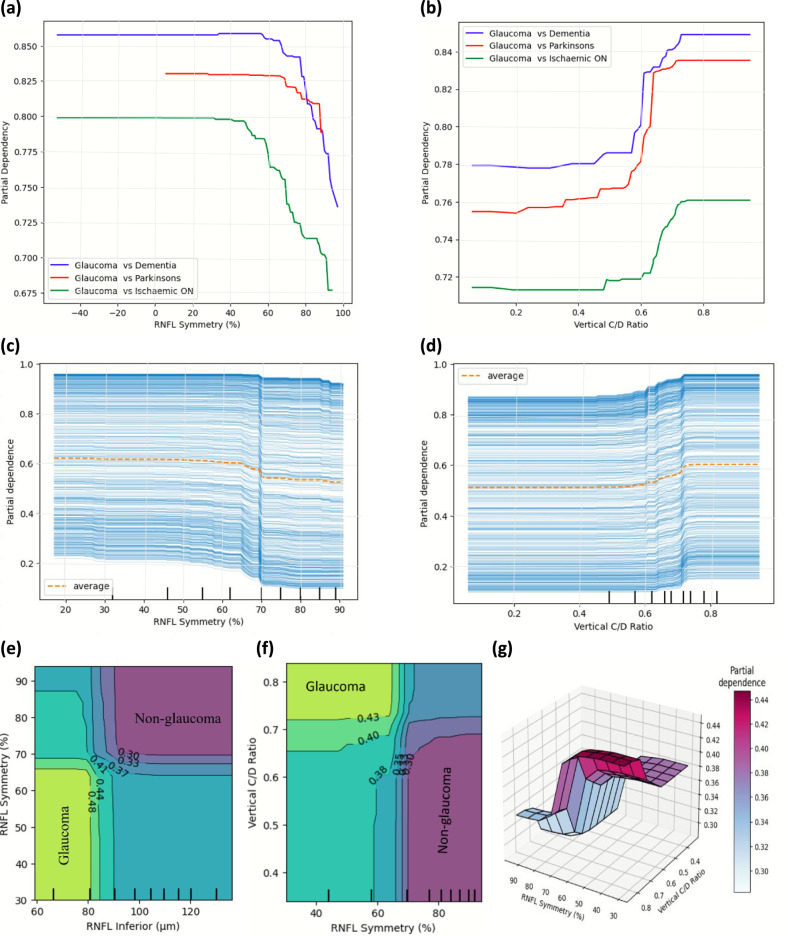
(**a**–**d**) Comparison of partial dependency plots (PDPs) for glaucoma versus normal, dementia, Parkinson’s, and ION individually (one versus one). (**e**–**g**) Numerical partial dependency plot (PDP) comparison between one-way and two-way analyses.

To understand how the values of features influence the predicted outcome of the classification model, several plots were simultaneously generated for the classifier distinguishing glaucoma from non-glaucoma cases (Fig. 4). The decision boundary for distinguishing glaucoma versus non-glaucoma groups (dementia, Parkinson’s, and ION) is depicted in the partial dependence plots (PDPs) (Fig. 4a–d) for the most important features ranked by SHAP analysis. The plots (Fig. 4a) sharply drop from left to right, indicating that lower symmetry corresponds to an increased likelihood of a glaucoma diagnosis, and revealing a clear decision boundary. Conversely, the plots (Fig. 4b) sharply rise from left to right for vertical C/D ratio, i.e., the higher the vertical C/D ratio, the higher the chances of glaucoma. Additionally, the descending slopes show different cut-offs in the decision boundaries for glaucoma versus dementia, glaucoma versus Parkinson’s, and glaucoma versus ION classifiers. This suggests that the secondary neurodegenerative conditions undergo some changes in OCT features such as higher RNFL asymmetry, reduced RNFL inferior thickness, and higher C/D ratio. However, the changes are slower and different from the primary condition, i.e., glaucoma. The individual conditional expectation (ICE) plots (Fig. 4c,d) show effect of individual subjects on the prediction, which combinedly produces a mean partial dependence (dashed line).

To gain a more comprehensive understanding, one-way (Fig. 4c,d) and two-way (Fig. 4e,f) dependency plots were generated for glaucoma diagnosis compared to other non-glaucoma cases (normal + dementia + Parkinson’s + ION) (Fig. 4c–d). A distinct descending slope is evident between 65–70% for RNFL symmetry (Fig. 4c), while there is an ascending slope of 0.60–0.75 for vertical C/D ratio (Fig. 4d) feature values.

In the two-way-dependency plots (Fig. 4e,f), a higher probability on the left indicates a greater chance of being classified as glaucoma, while a lower probability on the right signifies the likelihood of being classified as non-glaucoma. The plots also show how the predicted outcomes are affected by interactions among key features like RNFL symmetry and RNFL inferior thickness (Fig. 4c), and RNFL symmetry and vertical C/D ratio (Fig. 4d. The analysis revealed that below an RNFL inferior thickness of approximately 80 μm, the classifier mainly relies on RNFL symmetry, while beyond this threshold, it is influenced by both RNFL inferior thickness and RNFL symmetry combinedly, resulting in decreased probability of glaucoma classification (Fig. 4e). Furthermore, when RNFL symmetry surpasses 70% (vertical contour), it becomes largely independent of vertical C/D ratio, indicating a higher likelihood of non-glaucoma classification (Fig. 4f).

### Explainable ML-based app development

#### Non-private XML model

**Fig. 5:**
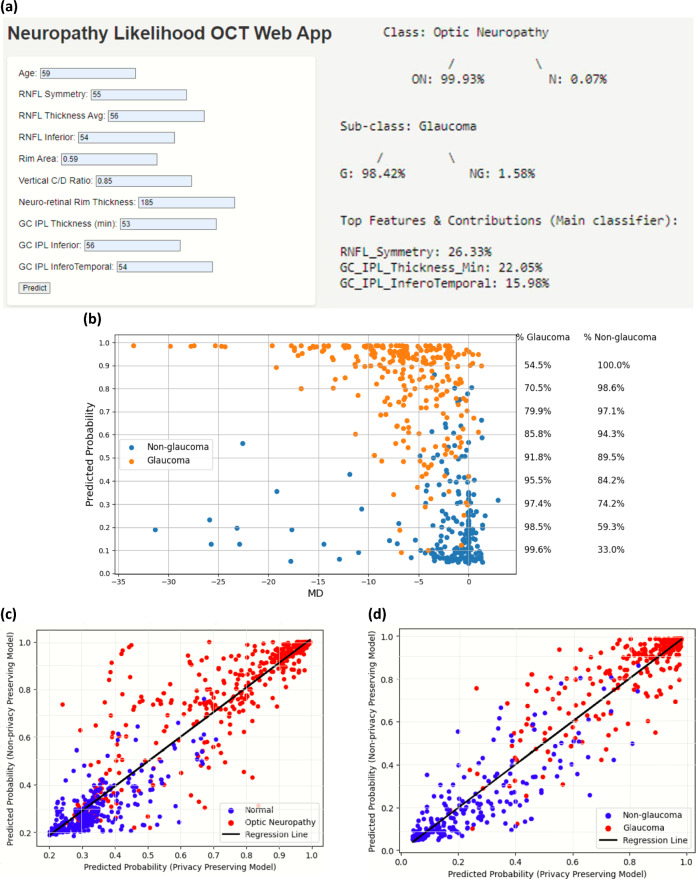
(**a**) Visual representation of the Neuropathy Likelihood OCT Web App featuring a web-deployed RF model. (**b**) Graph illustrating predicted probabilities for glaucoma in relation to the mean deviation of visual fields**.** Demographics: 196 glaucomatous eyes and 435 non-glaucomatous eyes (263 normal eyes, 35 dementia eyes, 45 Parkinson’s eyes and 92 ION eyes). (**c**) Correlation between privacy preserving model and non-privacy preserving model. for Classifier 1 (correlation coefficient = 0.94). (**d**) Correlation between privacy preserving model and non-privacy preserving model. for Classifier 2 (correlation coefficient = 0.95).

Based on the interpretable findings, a web-based tool named the Neuropathy Likelihood OCT App was created to aid clinicians in diagnosing neuropathy (Fig. 5a). The web application uses a reduced subset of the most explainable features identified via SHAP from the selected 82 features. Both classifiers were trained using the most crucial explainable features identified through SHAP analysis, encompassing a unified feature set for both classifications. Two RF models, one for Classifier 1 (optic neuropathy versus normal) and another one for Classifier 2 (glaucoma versus non-glaucoma), were deployed in the same app. If the test sample is identified as normal, only the first classifier is executed and the app will show the predicted probability of normal and neuropathy; otherwise, the second classifier will also be executed, to predict the probability of glaucoma or non-glaucoma. The tool enables users, particularly clinicians, to input the feature values. The application is built using Flask, a Python web framework, integrating HTML templates for interface with users, and operates within a client–server architecture, enabling clinicians to interact via a web browser while the server manages requests and responses.

To evaluate the model’s effectiveness, MD values were plotted against the predicted probabilities, which is the mean difference between the patient’s measured visual field sensitivity and the expected normal sensitivity for their age, expressed in decibels (dB) (Fig. 5b). It is a crucial measure for glaucoma diagnosis, which was also considered for glaucoma severity levels. For normal versus optic neuropathy, Classifier 1 yields 83.6% sensitivity, 94.9% specificity, and 0.901 AUC, while for glaucoma versus non-glaucoma, Classifier 2 yields 91.8% sensitivity, 89.5% specificity, and 0.914 AUC, at probability cut-off 0.5 (Fig. 5b).

The web application offers both predicted probabilities using global input features and local explanations through SHAP analysis for test samples, showcasing the top three contributing features and their percentages for classification. This empowers clinicians to scrutinise specific influential features like RNFL Symmetry and GC-IPL thickness, which may vary in importance across.

#### Privacy preserving XML model

As the feature set contains sensitive information (e.g., the patient’s age), we have developed a privacy-preserving explainable RF model with differential privacy mechanism. The model is trained with the same explainable feature sets as the non-private explainable model. However, it ensures privacy preservation, which adds value to the model. An epsilon value of 1 was used, representing the privacy budget controlling the trade-off between privacy and accuracy. For the differentially private model, the performance decreases from 0.901 to 0.876 AUC for Classifier 1 (other performance metrics: sensitivity 0.817, specificity 0.858, accuracy 0.826, F1 0.845) and from 0.914 to 0.896 AUC for Classifier 2 (other performance metrics: sensitivity 0.855, specificity 0.812, accuracy 0.836, F1 0.855), due to Laplacian randomness added to the input. Overall, the AUC reduction is 0.02 (Classifier 1)—0.03 (Classifier 2), sensitivity reduction is 0.01 (Classifier 1)—0.017 (Classifier 2), and specificity reduction is 0.017 (Classifier 1)—0.074 (Classifier 2). The correlation coefficient between the predicted probability of the non-privacy preserving and the privacy preserving classifier was 0.94 for Classifier 1 and 0.95 for Classifier 2 (Fig. 5c,d).

### Robustness testing against artefacts

The dataset used in this study contained RNFL, GC and MC thickness features. Among all the included patients, the data of 1.33% of them had artefacts that affect the optic disc while analysing and extracting those thickness features, as identified by three experienced clinicians. Of those, 0.85% cases were totally removed from the dataset used for the TSNIT Plots and subsequent ML-based analysis. However, 5.05% cases contained artefacts (such as truncation error and media opacities) affecting some of the measured features, while others were not affected. We included those cases in our dataset, but deleted the data points containing artefacts (e.g., GC and MC), while retaining the other data points (e.g., RNFL), and imputing the deleted (artefact prone) data points using MICE. Regarding partially missing data, it affected either 8 GC or 12 MC thickness features out of 132 total features (a list of all features are presented in Supplementary Table 1). Mostly GC and MC thickness features were affected for the 5.05% cases, which is around 15% of all features. To test the robustness of our model against artefacts in the data, a subanalysis was performed training the model using the partially artefactual data (clean data and 5.05% partially artefactual data with MICE imputation) and testing the model with fully artefactual data (0.85%), which were initially removed from the dataset (0.85%). Classifier 1 achieved 100% sensitivity, 90% specificity, 94.1% accuracy, 94.7% F1-score, and 0.95 AUC while using those 0.85% artefact containing (RNFL + GC + MC) samples as test set, which shows a strong robustness of the model to data containing artefacts. We also performed a stratified analysis comparing the performance of an RF classifier using (i) clean cases with no missing data, (ii) partially imputed cases (5.05%), and (iii) fully artifactual cases (0.85%). The results show that model performance remains stable across groups, with only minimal variation in AUC, sensitivity and specificity (Supplementary Table 5).

## Discussion

The present study demonstrates the feasibility of an XML-based approach for differential diagnosis of glaucoma and other neurodegenerative diseases. The study outcomes encourage discussion on four perspectives: diagnostic performance, research implications, study limitations, and potential avenues for future research. First, the study outperformed recent ML-based studies using OCT/OCT angiography (OCTA) and other modalities. Using CIRRUS OCT-derived retinal thickness data, our ML models achieved an AUC of 0.90 (95% CI 0.89–0.91) for distinguishing overall optic neuropathy from normal. If neuropathy, ML produced an AUC of 0.95 (95% CI 0.92–0.98), 0.96 (95% CI 0.94–0.98) and 0.91 (95% CI 0.85–0.97) while differentiating dementia, Parkinson’s, and ION from glaucoma. Manassakorn et al.^44^ used a deep learning model named GluNet trained with OCTA images of 258 glaucomatous and 439 non-glaucomatous eyes and the model was tested on 27 glaucomatous and 48 non-glaucomatous eyes, where they achieved 0.89 AUC. Similarly, Xiong et al.^45^ used OCT scans to differentiate glaucoma and non-glaucoma eyes (total 1,083 patients) and achieved 0.81 AUC using their OCTNet deep learning model. Conversely, we used only OCT spatial-frequency features and achieved 0.93 AUC (95% CI 0.90–0.95) using SVM for differentiating 268 glaucomatous and 209 non-glaucomatous eyes, which is higher than the aforementioned studies. The unique aspect of our study is that we included neurodegenerative conditions in the cohort in addition to non-glaucomatous ON, which the above studies did not. Second, our study demonstrated superior performance compared to clinicians across multiple trials in overall glaucoma diagnosis, surpassing sensitivity and specificity rates reported in studies by Rues et al.^46^ (accuracy 80.5%), Liu et al.^47^ (sensitivity 75.6% and specificity 77.8%), and Hedwin et al.^48^. While the latter observed a notable sensitivity–specificity discrepancy (18%) among optometrists, our study achieved high sensitivity (88.7% using SVM) and specificity (88.6% using RF, 86.1% using SVM) rates, addressing this disparity and achieving improved accuracy (92.7% using SVM) in glaucoma versus non-glaucoma classification.

Third, our study outperforms some recent ML based studies irrespective of usable modalities and classifiers while differentiating neurodegenerative diseases (plus other optic neuropathies) from normal and between themselves. For example, Wang et al.^49^ used OCT features for the diagnosis of dementia (159 patients) from normal (299 patients) and achieved a test AUC of 0.52–0.75, while testing with seven different supervised ML classifiers. Diaz et al.^50^ achieved an AUC of 0.69 and 0.71 using two separate datasets while differentiating Parkinson’s patients from normal using a SVM classifier. Fourth, our study showed comparable performance compared to the prior visual field-only studies that have demonstrated improved glaucoma classification using meta-learning or ensemble frameworks. For example, Moradi et al.^51^ analysed a subset of 33,636 participants (58% female) comprising 340,444 visual fields (VFs) using stacked weight-based machine learning approaches with three meta-learners: logistic regression (LR), extreme gradient boosting (XGB), and multilayer perceptron (MLP). Their objective was to distinguish glaucoma cases from non-glaucoma cases. Among the evaluated models, the MLP meta-learner delivered the best results, achieving 96.43% accuracy, a 96.01% F-score, and a 97.96% AUC. The meta-learning framework surpassed the performance of individual stand-alone models in glaucoma classification, improving accuracy by 8.92% compared with the best-performing stand-alone model, LoGTS, which achieved 87.51% accuracy. In our study, we achieved an AUC of 0.90 (95% CI 0.89–0.91) (RF) while classifying the combined set of dementia, Parkinson’s, ION and glaucoma patients (N = 419 eyes) against normal (N = 334 eyes). Also, we have performed those classifications and using our SVM model we achieved an AUC of 0.95 (95% CI 0.92–0.98), 0.96 (95% CI 0.94–0.98) and 0.91 (0.85–0.97) for classifying dementia, Parkinson’s and ION respectively against glaucoma. To the best of our knowledge, no recent study has experimented with classification of primary neurodegeneration (glaucoma) versus secondary neurodegeneration (dementia/Parkinson’s disease/ION), or intra-secondary neurodegeneration.

The interpretation of our models using explanatory methods aligns with the conclusions of most clinical studies. For example, Kara-Jose et al.^52^ have identified the correlation of different OCT features and disc damage likelihood scale (DDLS) due to glaucoma, and identified the strongest correlation with vertical cup-to-disc ratio (r = 0.71), followed by Rim Area (r = -0.64). A similar finding was obtained by Han et al.^53^, who observed the strongest correlation of DDLS with Rim area (r = -0.75), followed by vertical cup-to-disc ratio (r = 0.74) measured from CIRRUS OCT. Hong et al.^54^ found that the diagnostic effectiveness of interocular RNFL thickness symmetry (AUC = 0.96) surpassed that of RNFL thickness alone (AUC = 0.88) in early glaucoma. Additionally, Wu et al.^55^ highlighted the significant influence of RNFL inferior in the detection of glaucoma. In our investigation, the top ranked features identified by the SHAP analysis for glaucoma versus non-glaucoma are Rim Area and RNFL symmetry, followed by RNFL inferior thickness and vertical C/D ratio, which supports the findings of the above clinical studies.

This study stands out from previous ML research in glaucoma diagnosis by employing innovative techniques in feature engineering and neuropathy prediction, utilising frequency-domain features such as PSD and harmonics slope derived from a FFT of spatial domain TSNIT patterns, emphasising the importance of global frequency-domain measures in diagnosis. The local feature importance determined through SHAP analysis during the inference phase empowers clinicians to reevaluate and examine specific features that have a substantial impact on the classification or diagnosis. This provides clinicians with confidence and trust in the results generated by artificial intelligence. However, several points must be considered when interpreting these findings. Firstly, the model’s applicability is currently confined to specific OCT systems (i.e., CIRRUS and HFA) and not universally adaptable to all OCT machines. Secondly, the study encountered data imbalance issues due to the removal of artefacts, resulting in a smaller glaucoma cohort in comparison to non-glaucoma patients, which was addressed using SMOTE-based oversampling^27^. Furthermore, missing data in different OCT parameters led to the utilisation of the MICE algorithm^17^ for imputation, which is a potential solution given real-world data often contains artefacts. The feature selection was done separately on the full-dataset (clean data without imputation) using the ANOVA F test, correlation and then a wrapper method with cross-validation, to finally arrive at a minimal explainable feature set for the app. As we did not use the same cross-validation for classification, this approach might be prone to optimistic bias. Future work could involve feature selection within each cross-validation fold. Third, the SHAP and PDA interpretation techniques are computationally expensive, although a user-friendly offline tool can be developed once feature probabilities are predicted using PDA. Regarding privacy preservation, although we have used a differential privacy scheme, other privacy preserving approaches such as (differential) federated learning may enhance the security of the ML model. Fourth, diagnosis of dementia and Parkinson’s disease was based on self-report and medication history, which may introduce diagnostic uncertainty. Future work will incorporate verified clinical records, including diagnosis using magnetic resonance imaging (MRI) and/or positron emission tomography (PET). Fifth, exclusion of patients with co-existing glaucoma and neurodegenerative disease may limit real-world applicability. Future studies will incorporate multimorbidity cohorts. Future enhancements could also involve the integration of the proposed tool into a web-based app for real-time clinical use, the inclusion of additional clinical features^56,57^, and the differentiation of glaucoma suspects from normal cases, possibly through multimodal data fusion, such as colour fundus images with OCT features to bolster the diagnostic accuracy.

## Supplementary Information

Supplementary Information.

## Data Availability

The patients provided informed consent and approval for data to be used as part of our study (UNSW human research ethics approval number: HC210563). Consequently, the raw data are not available for public access, but the statistically analysed OCT data in tabular format for a group of patient cohorts (normal, glaucoma, ischaemic optic neuropathy, dementia and Parkinson’s disease) may be accessible upon reasonable request to the corresponding author.
