# Supplementary material for: OCT-based optic neuropathy diagnosis using explainable and privacy-preserving machine learning
Source: Sci Rep. 2026 May 26;16:24042. doi: 10.1038/s41598-026-53687-x (PMC13439056; doi:10.1038/s41598-026-53687-x)
Supplement: Supplementary file 1 [file 41598_2026_53687_MOESM1_ESM.pdf]

Supplementary Material

OCT-based optic neuropathy diagnosis using explainable and privacy-preserving machine learning

Md Mahmudul Hasan[a,*], Jack Phu[b,c,d,e], Henrietta Wang[b,c,e], Michael Kalloniatis[b,e,f], Erik Meijering[a], Arcot Sowmya[a]

[a] School of Computer Science and Engineering, University of New South Wales, Sydney, NSW 2052, Australia
[b] School of Optometry and Vision Science, University of New South Wales, Sydney, NSW 2052, Australia
[c] Centre for Eye Health, University of New South Wales, Sydney, NSW 2052, Australia
[d] Faculty of Medicine and Health, University of Sydney, Camperdown, NSW, Australia
[e] School of Medicine (Optometry), Deakin University, Waurn Ponds, VIC, Australia
[f] University of Houston College of Optometry, University of Houston, Houston, TX, United States of America

[*] *Corresponding author*
Md Mahmudul Hasan
School of Computer Science and Engineering
University of New South Wales, Sydney, NSW 2052, Australia
md_mahmudul.hasan@unsw.edu.au

Short title: Optic neuropathy diagnosis using private explainable machine learning

Commercial Relationship disclosure: Md Mahmudul Hasan, Jack Phu, Henrietta Wang, Arcot Sowmya, Michael Kalloniatis, Erik Meijering have submitted a patent application (#2024900616) in the name of "NewSouth Innovations Pty Limited" and "Deakin University" which is currently lapsed. They declare that they have no known competing financial interests or personal relationships that could have appeared to influence the work reported in this paper.

Keywords: optical coherence tomography, glaucoma, ischaemic optic neuropathy, dementia, Parkinson's disease, perimetry, explainable machine learning, SHAP analysis, partial dependency analysis

Supplementary Tables

Supplementary Table 1: *List of features extracted in spatial and frequency domain*

**RNFL thickness Analysis**	**Count**	**GC-IPL thickness Analysis**	**Count**	**Macular thickness Analysis**	**Count**
RNFL symmetry (%)	1	Average GC-IPL thickness	1	ILM-RPE thickness-central subfield	1
Average RNFL thickness (µm)	1	Minimum GC-IPL thickness (µm)	1	ILM-RPE thickness-volumetric cube	1
RNFL thickness ring area (mean) (µm)	1	GC-IPL superior (µm)	1	ILM-RPE thickness-average cube	1
RNFL thickness in quadrants (µm)		GC-IPL superonasal (µm)	1	ILM-RPE centre-foveal	1
• RNFL superior (mean, med, std, skew & kurt)	5	GC-IPL inferonasal (µm)	1	ILM-RPE inner superior (iSup)	1
• RNFL nasal (mean, med, std, skew & kurt)	5	GC-IPL inferior (µm)	1	ILM-RPE inner nasal (iNas)	1
• RNFL inferior (mean, med, std, skew & kurt)	5	GC-IPL inferotemporal (µm)	1	ILM-RPE inner inferior (iInf)	1
• RNFL temporal (mean, med, std, skew & kurt)	5	GC-IPL-superotemporal (µm)	1	ILM-RPE inner temporal (iTem)	1
RNFL clock hours				ILM-RPE outer superior (oSup)	1
RNFL clk 1- 12	12			ILM-RPE outer nasal (oNas)	1
RNFL 256 data points (0 to 256)				ILM-RPE outer inferior (oInf)	1
• Median	1			ILM-RPE outer temporal (oTem)	1
• Standard deviation	1				
• Skewness	1				
• Kurtosis	1				
Rim area	1				
Disc area	1				
Average cup to disc ratio	1				
Vertical cup to disc ratio	1				
Cup volume	1				
Neuro-retinal Rim Thickness	1				
TSNIT Shannon entropy (information theory)	1				
TSNIT Fisher information (information theory)	1				
TSNIT Signal to noise ratio (SNR) (information theory)	1				
FFT absolute value (real + imaginary)	1				
FFT real value	1				
FFT imaginary value	1				
PSD whole TSNIT (mean, maximum, median, standard deviation, skewness & kurtosis)					
FFT Method	6				
Welch Method	6				
Slope of PSD of TSNIT in (1st-4th) harmonics					
• FFT method	4				
• Welch method	4				

**RNFL thickness Analysis**	**Count**	**GC-IPL thickness Analysis**	**Count**	**Macular thickness Analysis**	**Count**
	1				
Slope of PSD of TSNIT (mean, maximum and minimum) (FFT & Welch)	6				
PSD of RNFL superior quadrant (mean & maximum) (FFT & Welch)	4				
PSD of RNFL temporal quadrant (mean & max) (FFT & Welch)	4				
PSD of RNFL nasal quadrant (mean & maximum) (FFT & Welch)	4				
PSD of RNFL inferior quadrant (mean & max) (FFT & Welch)	4				
PSD of TSNIT with level-(1~4) DWT (mean & max)					
• FFT method	8				
• Welch method	8				
Spectral entropy of PSD of TSNIT (information theory)	2				
Count (individual analysis)	112		8		12
Total Count	132				

Note: Med: median, std: standard deviation, skew: skewness, kurt: kurtosis, FFT: Fast Fourier Transform, DWT: Discrete Wavelet transform, PSD: power spectral density ('Welch' and FFT method), max: maximum value, DWT: discrete wavelet transforms ('Haar' wavelet), spatial domain features are above horizontal line, below the line are frequency domain features

Supplementary Table 2: *Hyperparameters considered for optimisation of the different classifiers*

**Classifier**	**Hyperparameter**	**Optimised Value (Primary Classifier)**	**Optimised Value (Secondary Classifier)**
KNN	▪ Number of neighbours (k) [1–40]	▪ K = 25	▪ K = 9
SVM	▪ 'C' value (0.1, 1, 10, 100, 1000)	▪ C = 10	▪ C = 100
	▪ Gamma (with 'RBF' karnel) (1, 0.1, 0.01, 0.001, 0.0001)	▪ Gamma = 0.01	▪ Gamma = 0.0001
RF	▪ Number of trees in the forest (n_estimate) [50, 100, 200, 300, 400]	▪ n_estimate = 300<br>▪ max_depth = 4	▪ n_estimate =100<br>▪ max_depth = 2
	▪ Maximum depth of the tree (max_depth) [2, 3, 4, 5, 6]		

Supplementary Table 3*: Features selected from the spatial and frequency domains.*

Feature rank	Spatial domain features	F-value	Feature rank	Frequency domain features	F-value
1	RNFL symmetry (percentage)	275.5	1	PSD slope-1(FFT)	169.6
2	GC-IPL thickness (minimum)	274.8	2	FFT absolute	112.2
3	GC-IPL inferotemporal	251.7	3	PSD slope-3 (FFT)	99.8
4	RNFL inferior (mean)	227.5	4	PSD of DWT TSNIT with level-4 ((Mean)	97.2
5	RNFL inferior (median)	221.9	5	PSD (Welch) of DWT TSNIT with level-4 (Max)	91.2
6	GC-IPL inferior (mean)	209.3	6	PSD (Welch) of DWT TSNIT with level-3 (Mean)	87
7	RNFL clock 6	205	7	Slope-2 of PSD (Welch) of TSNIT	82.8
8	RNFL clock 7	185.7	8	Spectral Entropy of TSNIT PSD (Welch)	82.6
9	CG-IPL thickness (average)	172.3	9	PSD (Welch) of DWT TSNIT with level-2 (Mean)	81.1
10	ILM-RPE outer inferior (oInf)	170.1	10	PSD (Welch) of DWT TSNIT with level-3 (Max)	79.8
11	TSNIT (std)	154.6	11	PSD (Welch) of TSNIT with level-1 (Mean)	78.3
12	Rim Area	150.9	12	PSD mean (Welch)	76.9
13	TSNIT kurtosis	149.5	13	PSD (Welch) of DWT TSNIT with level-2 (Max)	74.1
14	RNFL thickness (average)	136.4	14	PSD (Welch) of DWT TSNIT with level-1 (Max)	71.5
15	ILM-RPE thickness Avg Cube	129.5	15	PSD max (Welch)	70.2
16	GC-IPL superotemporal	112.7	16	PSD Slope-2 (FFT)	67.4
17	Vertical C/D Ratio	108.2	17	PSD slope-minimum (Welch)	64.3
18	ILM-RPE inner inferior (iInf)	106.9	18	TSNIT inferior maximum PSD (Welch)	61.7
19	ILM-RPE inner temporal (iTem)	102.5	19	PSD slope-maximum (Welch)	53.8
20	RNFL inferior (std)	100.4	20	TSNIT inferior mean PSD (Welch)	52.4
21	TSNIT median	98.7	21	PSD skewness (FFT)	47.8
22	GC-IPL inferoonasal	96.4	22	PSD slope-1 (Welch)	44.9
23	RNFL clock 11	95.1	23	PSD slope-mean (Welch)	33.8
24	RNFL superior (mean)	91	24	PSD slope-5 (Welch)	33.2
25	ILM-RPE outer temporal (oTem)	89.4	25	Spectral Entropy of TSNIT PSD (FFT)	31.9
26	GC-IPL superior	89.1	26	TSNIT superior maximum PSD (Welch)	31.6
27	RNFL superior (mean)	76.8	27	TSNIT inferior mean PSD (Welch)	29.3
28	SNR (dB)	74.3	28	PSD slope-5 (FFT)	27.7
29	Neuro-retinal Rim Thickness	69.4	29	PSD skewness (Welch)	24.2
30	ILM-RPE outer superior (oSup)	69.3	30	PSD slope-4 (FFT)	20.3
31	RNFL clock 12	64.4	31	TSNIT nasal maximum PSD (Welch)	20.1
32	Average C/D_Ratio	61.7			
33	RNFL clock 5	61			
34	RNFL thickness (ring scan)	60.8			
35	GC-IPL superonasal	57.4			
36	ILM-RPE outer nasal (oNas)	56.4			
37	ILM-RPE inner superior (iSup)	50.7			
38	ILM-RPE inner nasal (iNas)	43.2			
39	RNFL temporal (kurtosis)	42.5			
40	RNFL nasal (kurtosis)	37.7			
41	RNFL nasal (std)	37.3			
42	RNFL clock 2	36.7			
43	RNFL nasal (skewness)	34.9			

Feature rank	Spatial domain features	F-value	Feature rank	Frequency domain features	F-value
44	Cup Volume	28.5			
45	RNFL temporal (skewness)	27.2			
46	RNFL superior (std)	23.7			
47	RNFL inferior (kurtosis)	21.8			
48	TSNIT (skewness)	21.2			
49	RNFL clock 1	20.1			
50	RNFL temporal (std)	16.5			
51	Age	13			

Note: Med: median, std: standard deviation, FFT: Fast Fourier Transform, DWT: Discrete Wavelet transform, PSD: power spectral density ('Welch' and FFT method), DWT: discrete wavelet transforms ('Haar' wavelet)

Supplementary Table 4.: *Performance of clinicians in diagnosing glaucoma and neurodegenerative diseases. Boldfaced numbers indicate the highest sensitivity, specificity and accuracy achieved by the clinicians in a diagnosis.*

**Decision Maker**	**Metrics**	**Neuropathy vs Normals**	**If Neuropathy, Glaucoma vs Non-glaucoma**
Clinician 1 (Masked)	Sensitivity	**0.623**	0.397
	Specificity	0.563	0.538
	Accuracy	0.604	0.436
Clinician 2 (Masked)	Sensitivity	0.620	**0.796**
	Specificity	0.625	0.448
	Accuracy	**0.621**	0.688
Clinician 3 (Masked)	Sensitivity	0.513	0.793
	Specificity	**0.687**	**0.631**
	Accuracy	0.573	**0.753**
Mean Clinician Performance (Masked)	Sensitivity	0.585	0.662
	Specificity	0.625	0.539
	Accuracy	0.599	0.626
Clinician 1 (Unmasked)	Sensitivity	0.574	0.557
	Specificity	0.687	0.583
	Accuracy	0.614	0.565
Clinician 2 (Unmasked)	Sensitivity	**0.680**	**0.772**
	Specificity	**0.775**	0.666
	Accuracy	0.713	0.735
Clinician 3 (Unmasked)	Sensitivity	**0.680**	0.671
	Specificity	0.687	**0.947**
	Accuracy	**0.682**	**0.774**
Mean Clinician Performance (Unmasked)	Sensitivity	0.645	0.667
	Specificity	0.716	0.732
	Accuracy	0.670	0.691

Supplementary Table 5*: Comparative performance on clean data, partially artifactual data and fully artefactual data using a RF classifier.*

	Optic Neuropathy Versus Normal					Glaucoma Versus Non-glaucoma				
**Data**	**Sensitivity**	**Specificity**	**Accuracy**	**AUC**	**F1-Score**	**Sensitivity**	**Specificity**	**Accuracy**	**AUC**	**F1-Score**
Clean data	0.893	0.833	0.882	0.938	0.925	0.882	0.876	0.891	0.944	0.901
0.85% full artificial cases	1	0.901	0.941	0.951	0.947	0.901	0.815	0.872	0.955	0.891
5.05% partially imputed cases	0.825	0.873	0.872	0.951	0.918	0.875	0.833	0.867	0.934	0.915

Supplementary Figures

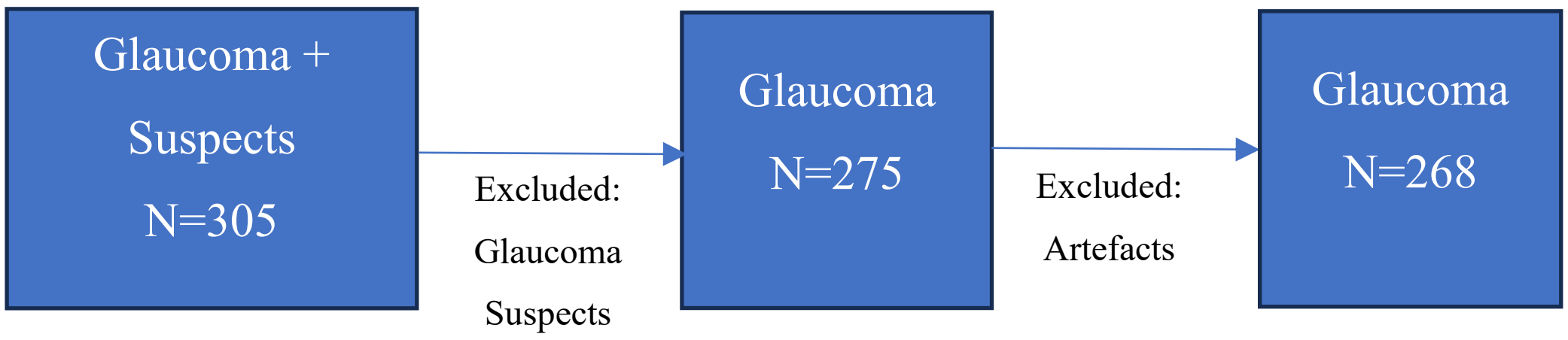

Supplementary Figure 1(a): *Mapping of the glaucoma patients cohort included in the study for differential diagnosis.*

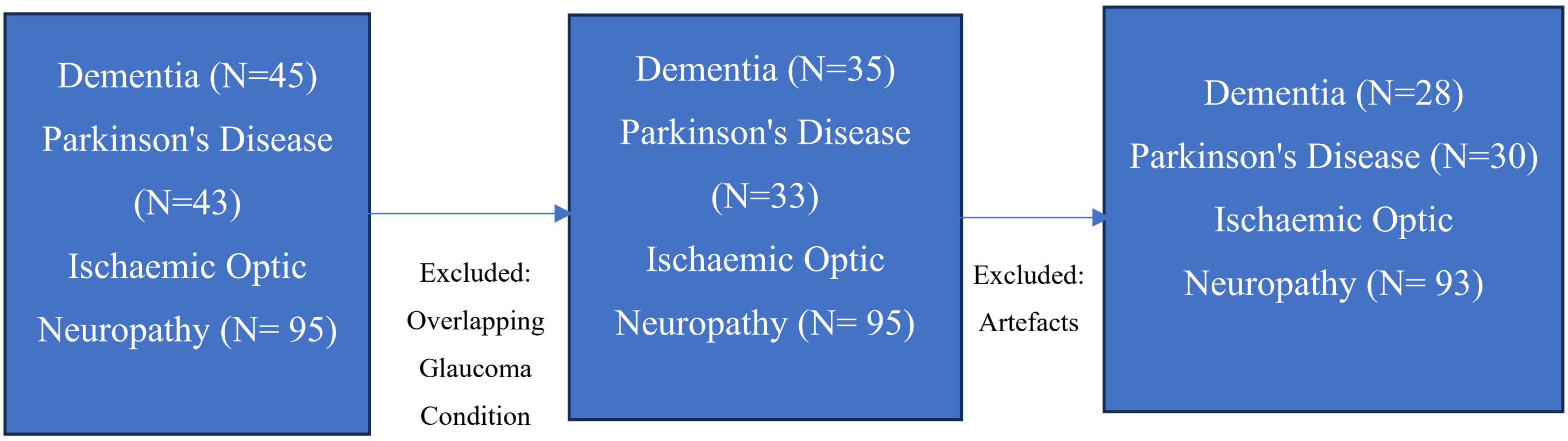

Supplementary Figure 1(b): *Mapping of the neurodegenerative patients cohort included in the study for differential diagnosis.*

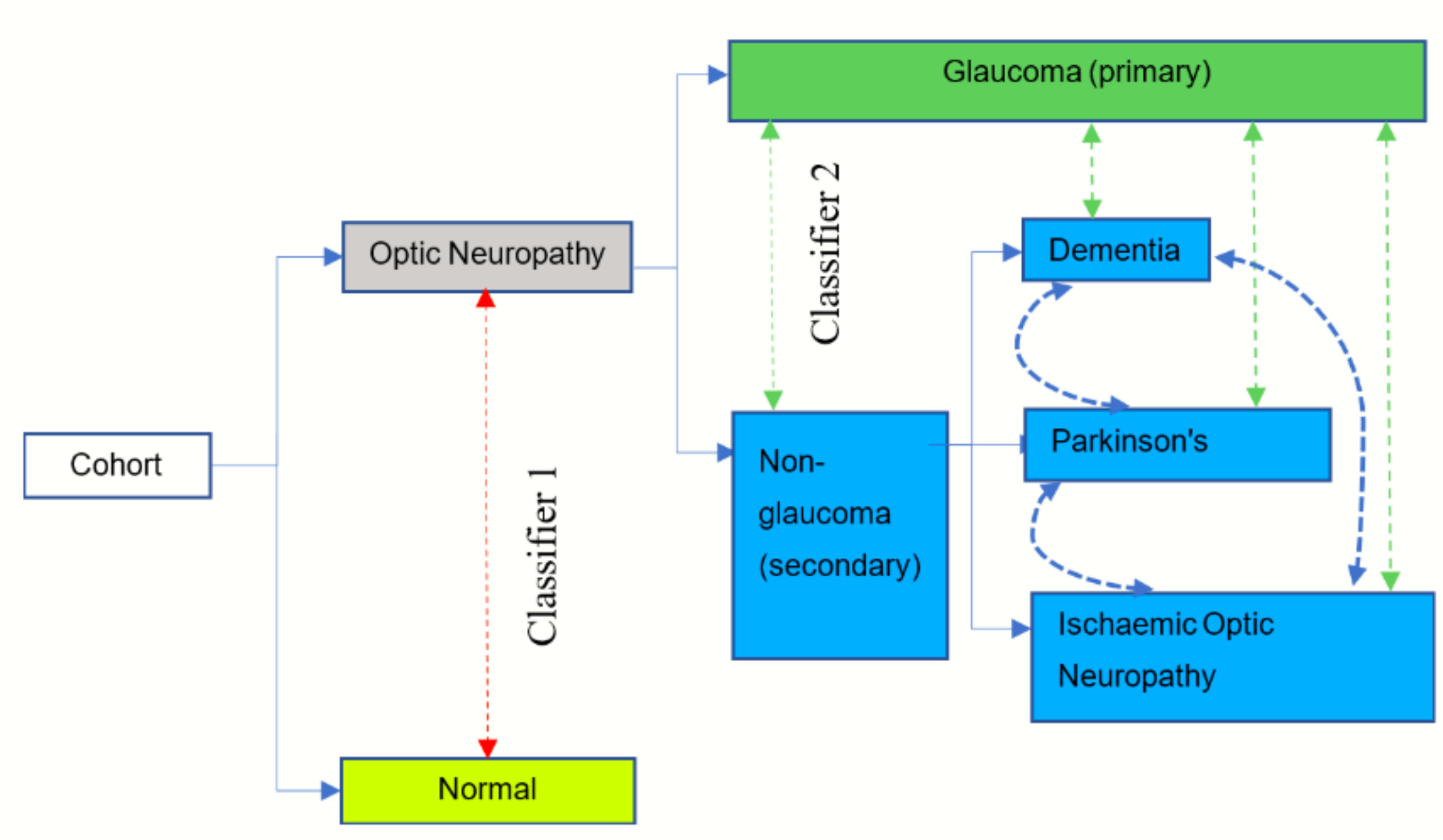

Supplementary Figure 1(c): *Mapping of the patient cohort included in the study for differential diagnosis using the two proposed classifiers. The green dashed lines represent sub-classifiers for glaucoma vs the secondary conditions (glaucoma versus dementia, glaucoma versus Parkinson's disease, and glaucoma versus ION), and the blue dashed lines represent sub-classifiers intra- secondary conditions (ION versus dementia, ION versus Parkinson's, and dementia versus Parkinson's).*

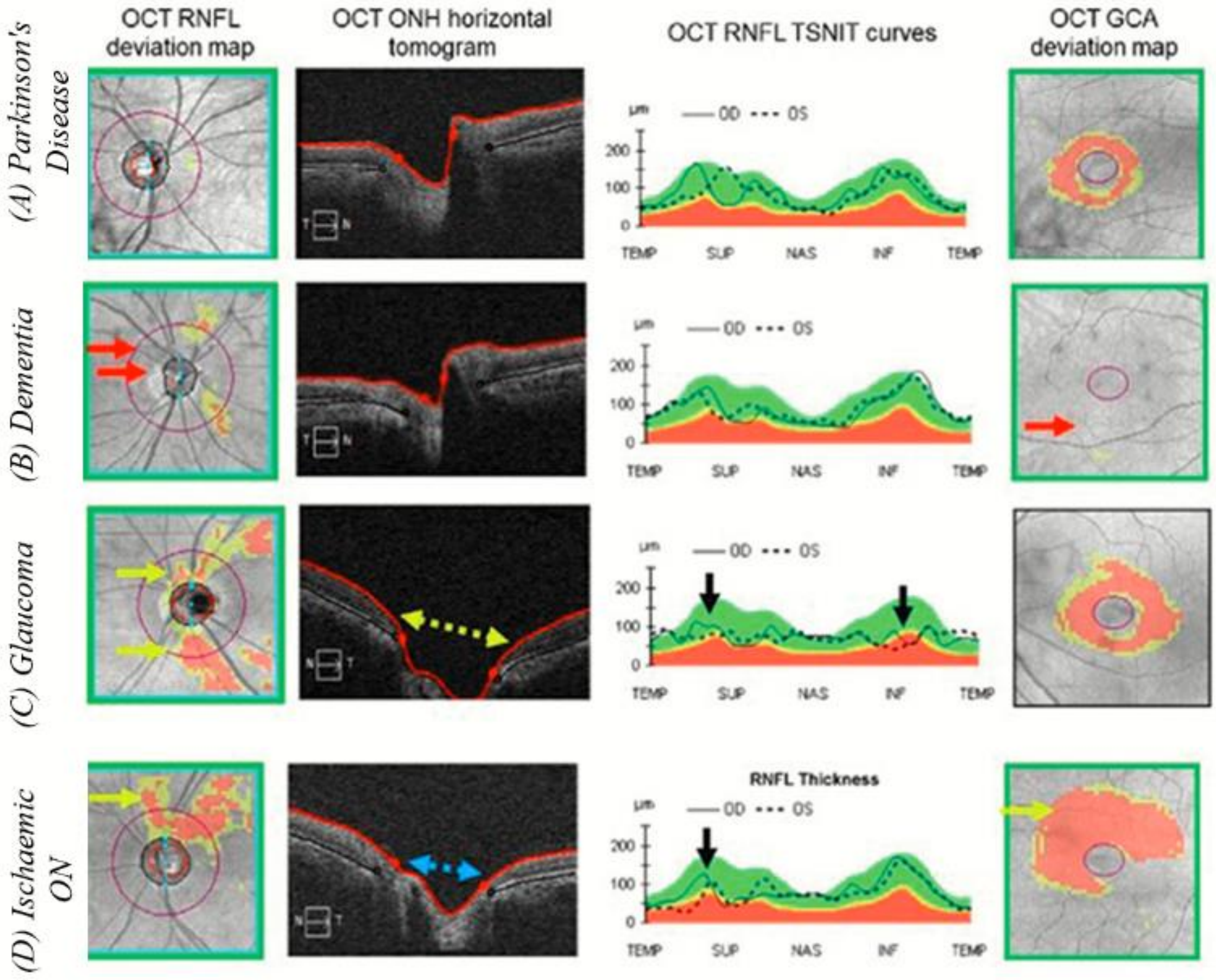

Supplementary Figure 1(d): *Example OCT images of each class, extracted from CFEH data. More detailed explanations can be found in Hasan et al (2024)*[1].

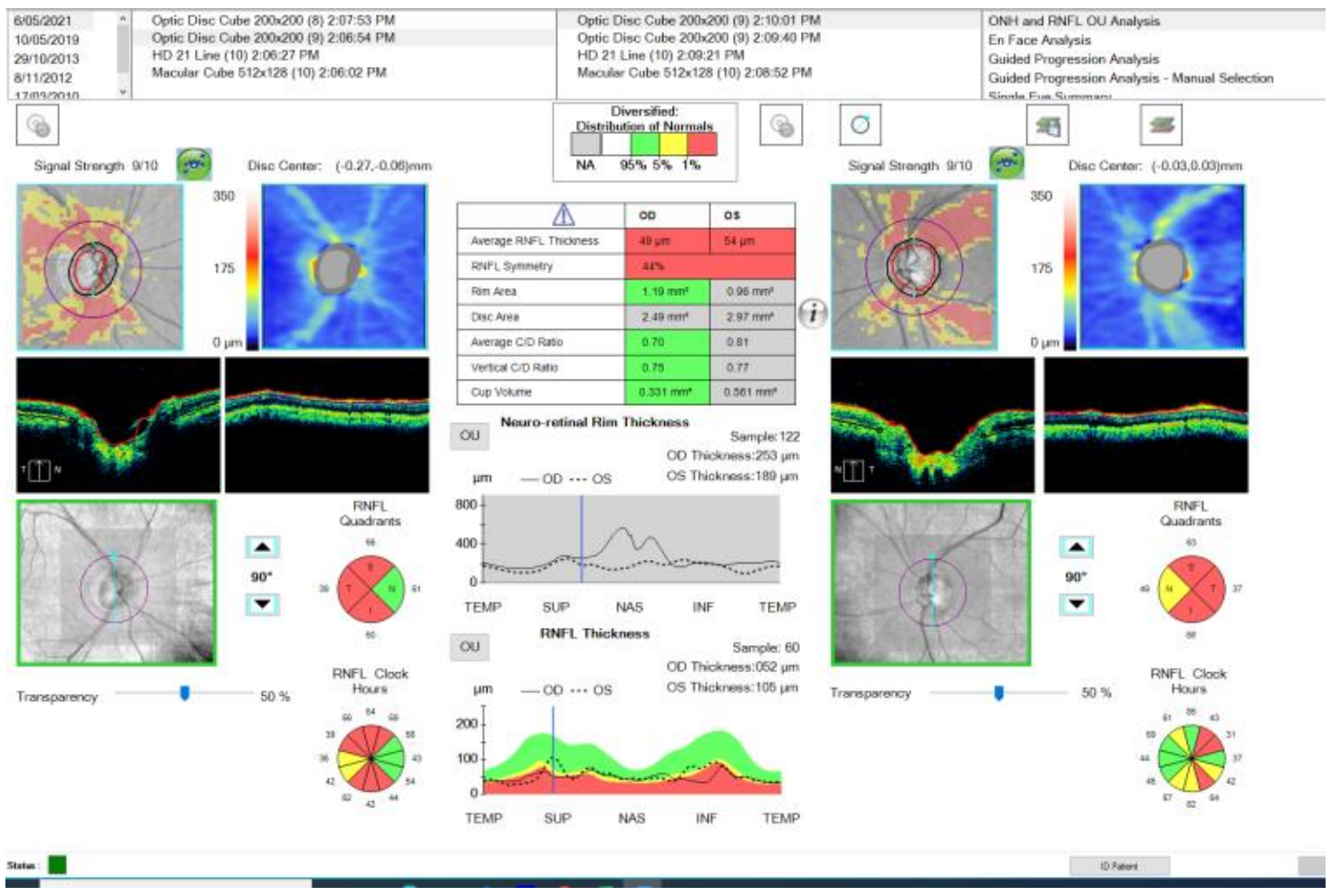

(a)

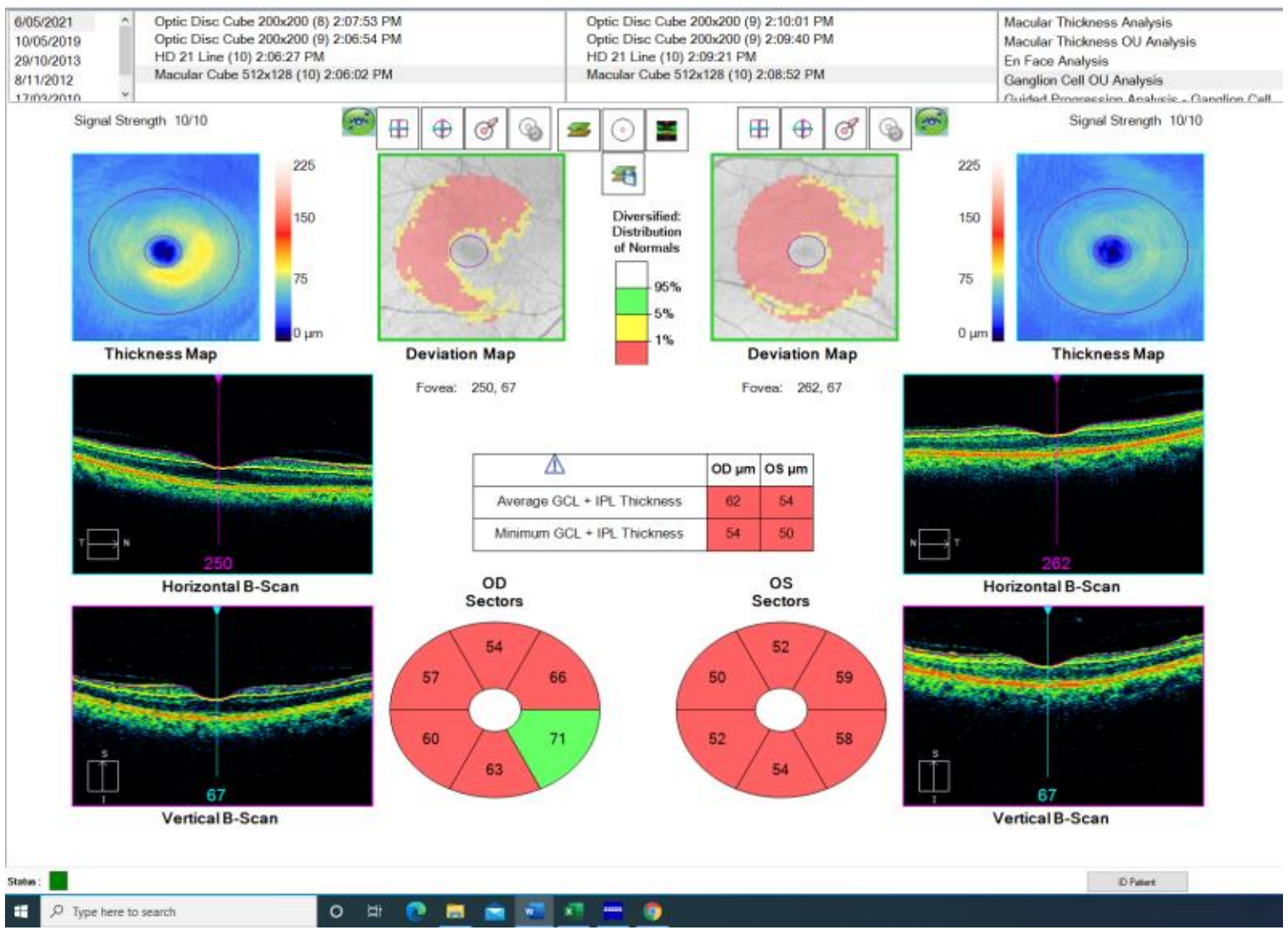

(b)

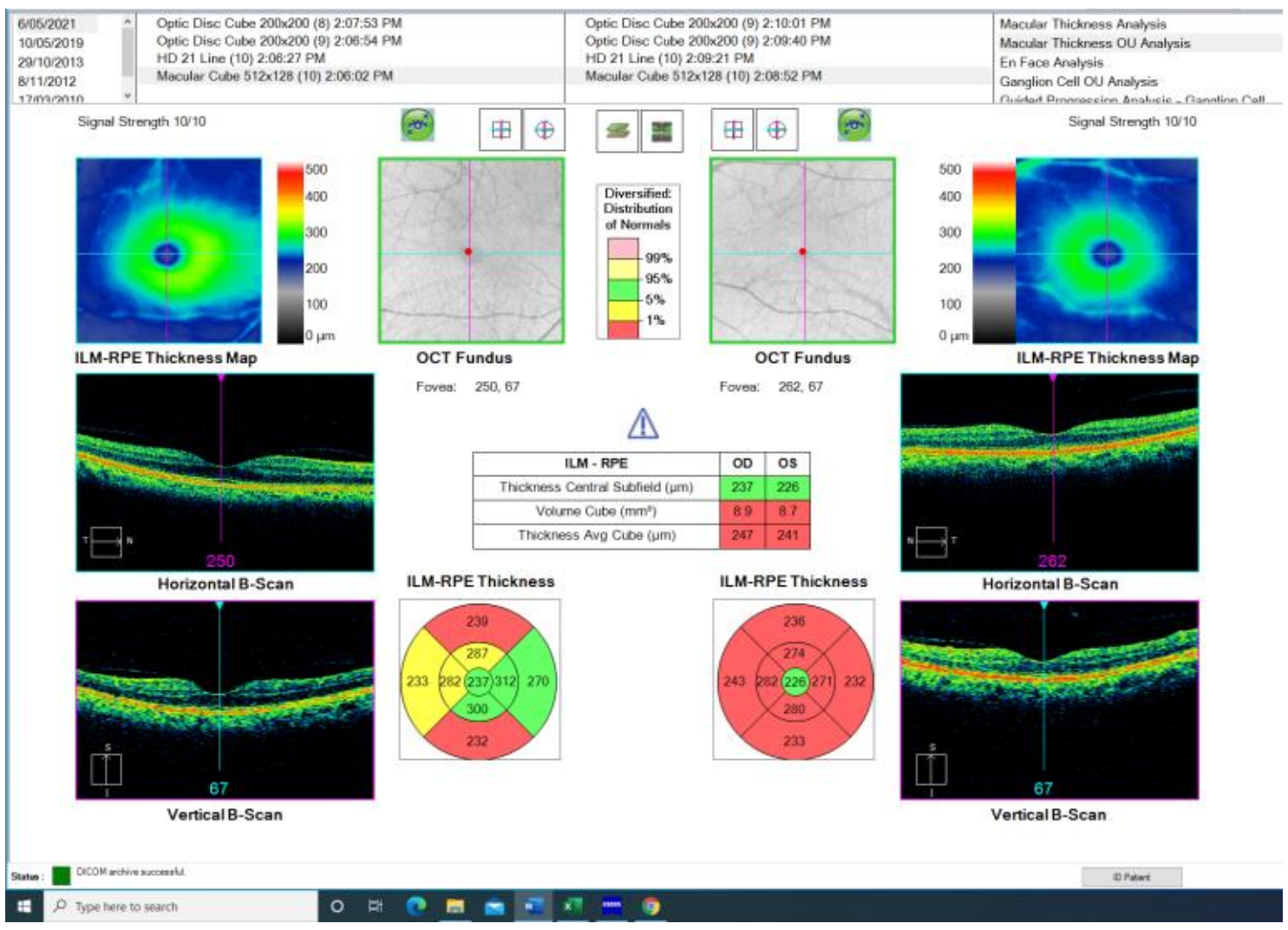

(c)

Supplementary Figure 2 (a): *Example of CIRRUS OCT based analysis (a) RNFL (b) GC-IPL (c) MC thickness analysis*

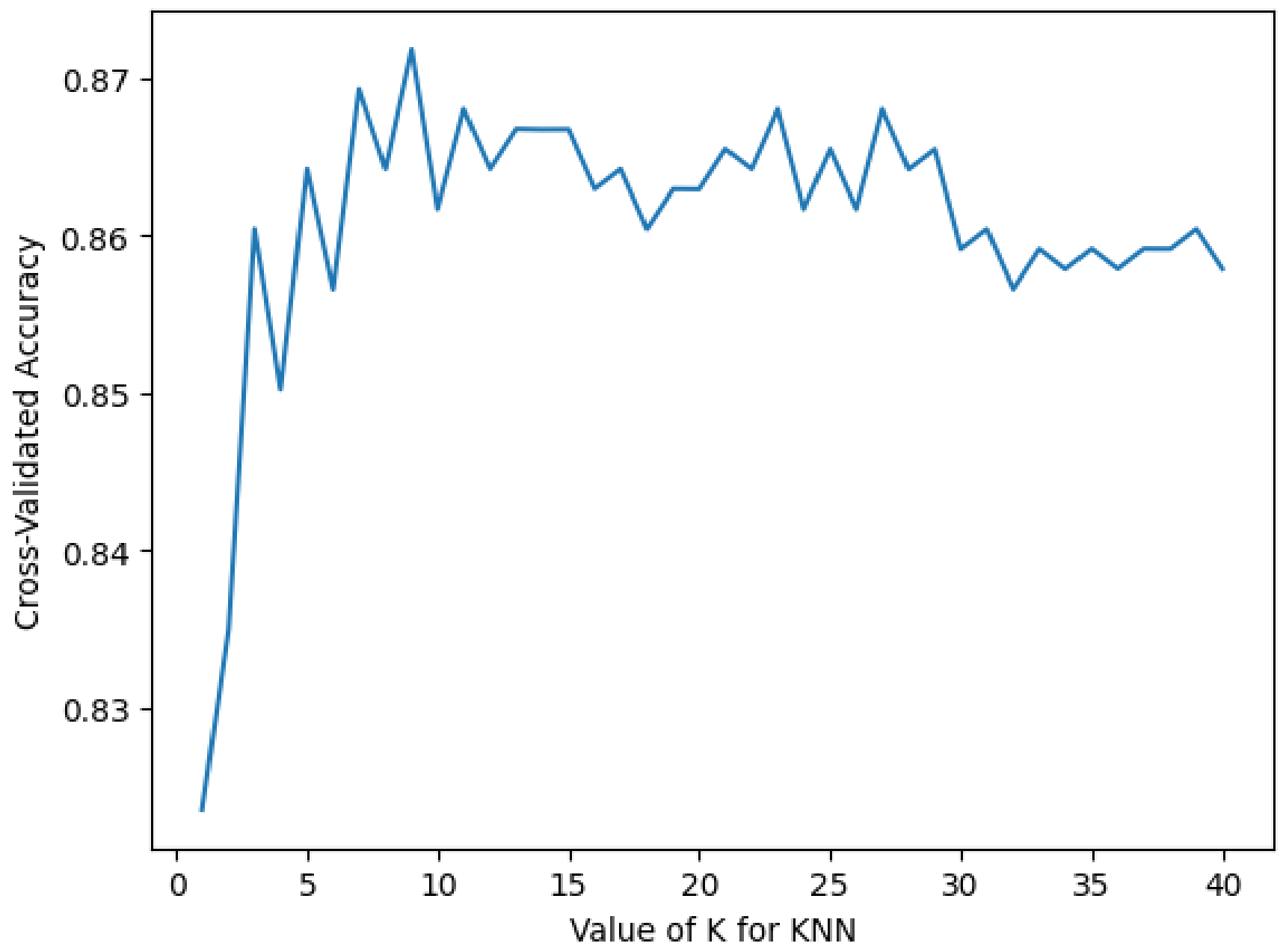

Supplementary Figure 3: *Hyperparameter tuning for KNN (example of Glaucoma overall vs non-glaucoma classification, value of k varied between 1 to 40, optimal number of neighbours, k=9)*

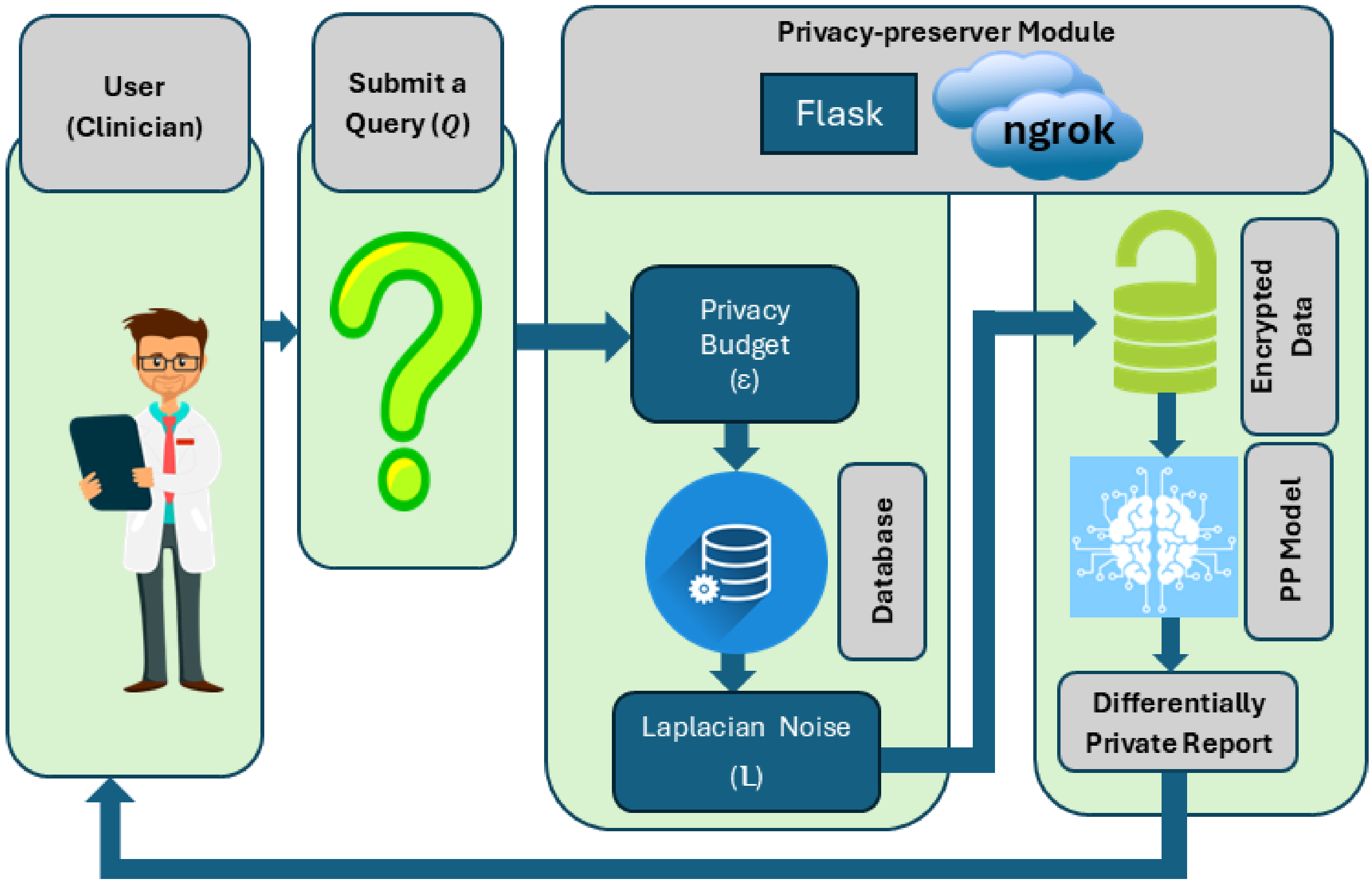

Supplementary Figure 4: *Flow-chart of the implementation of the diagnostic tool (classifier 1 & 2) based on XML (private).*

(a) RE TSNIT plot

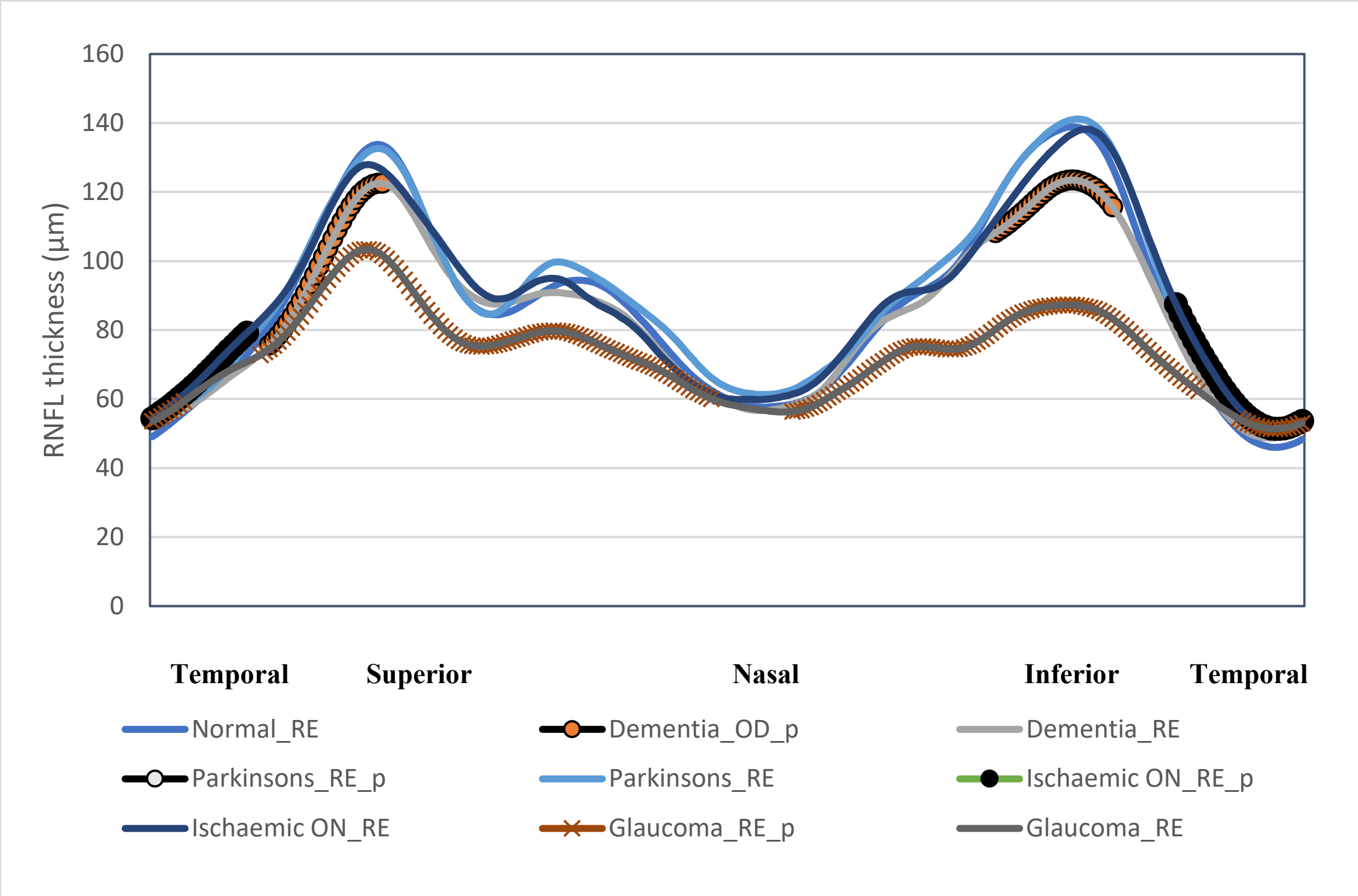

(b) LE TSNIT plot

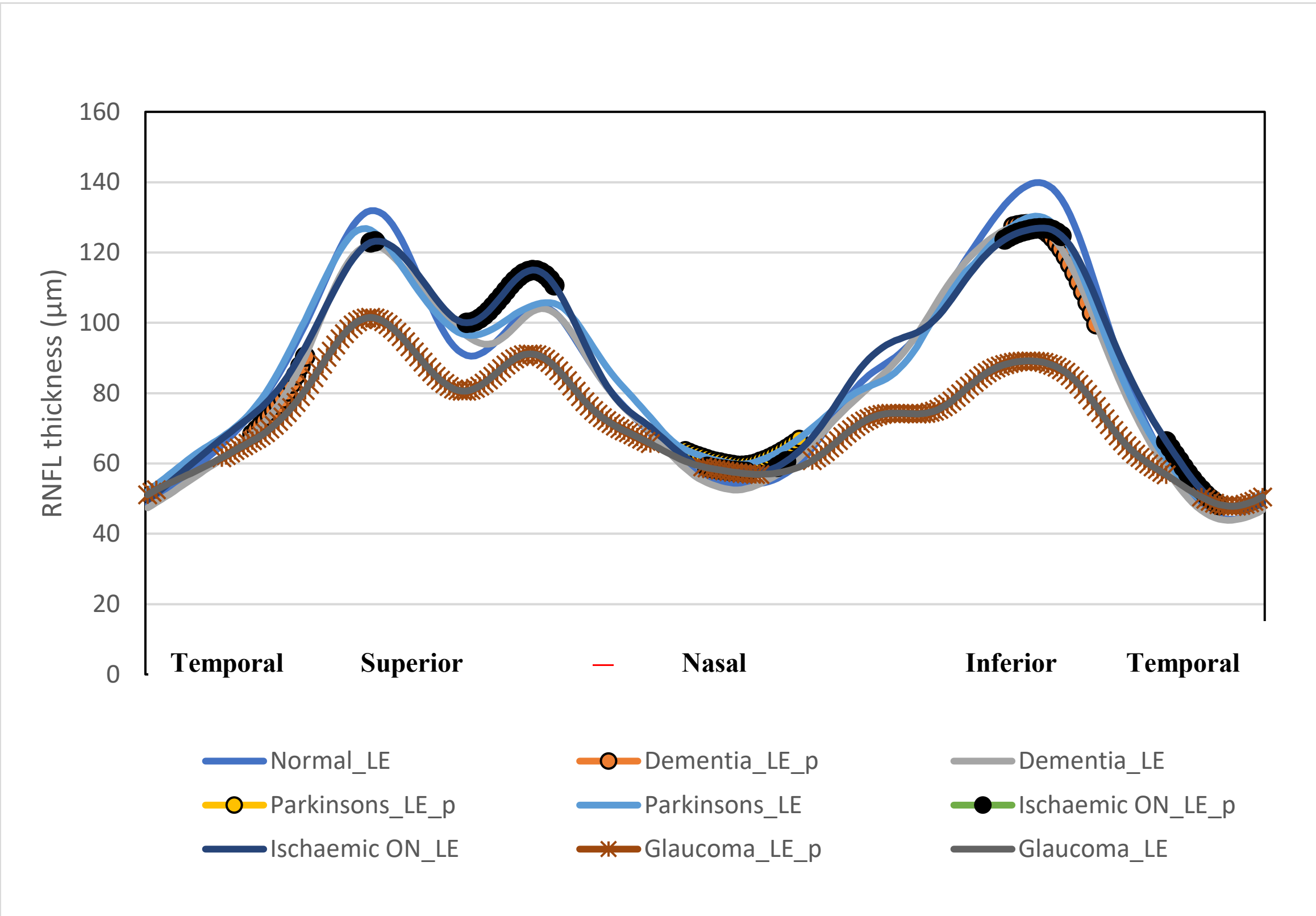

(c) RE difference plot

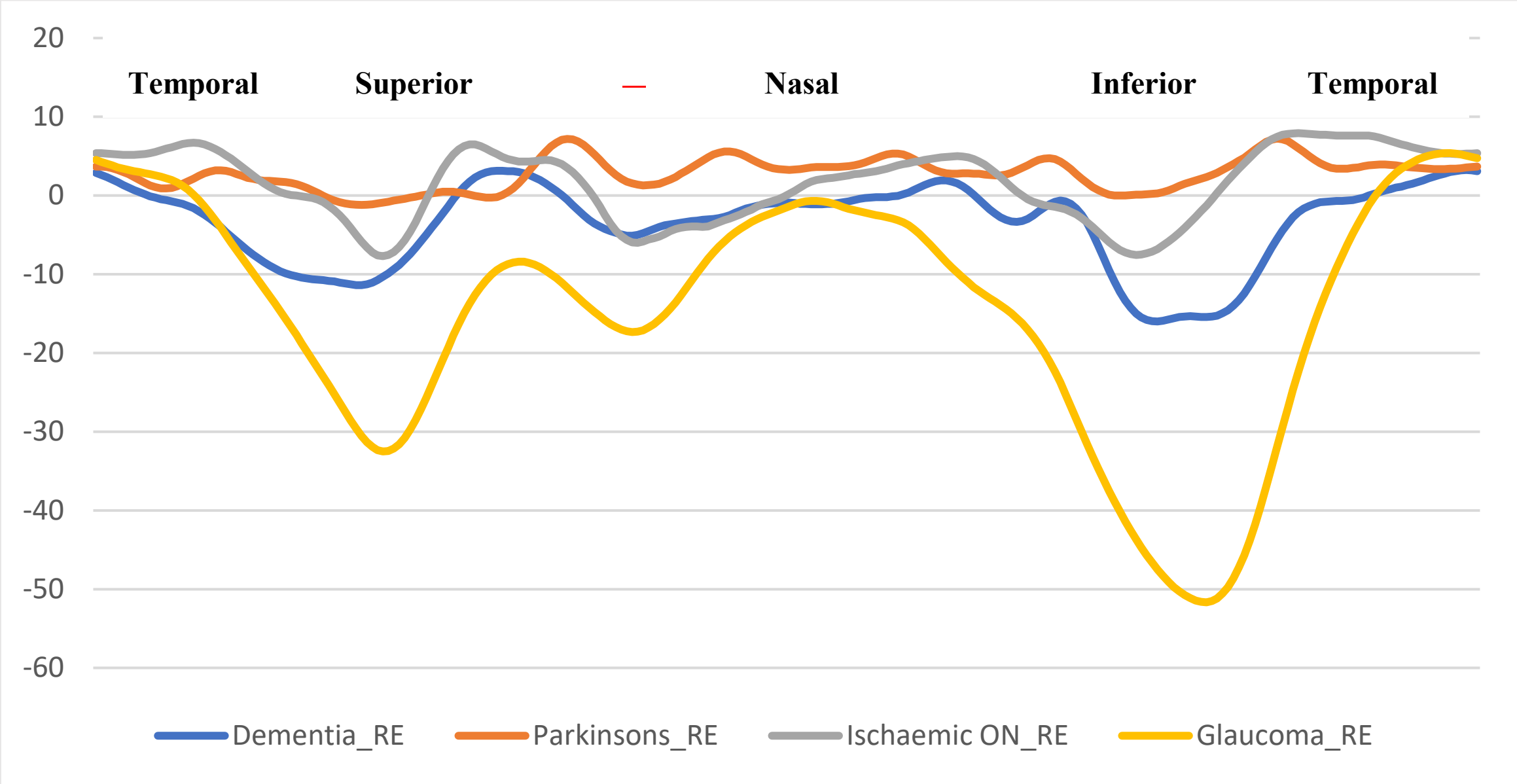

(d) LE difference plot

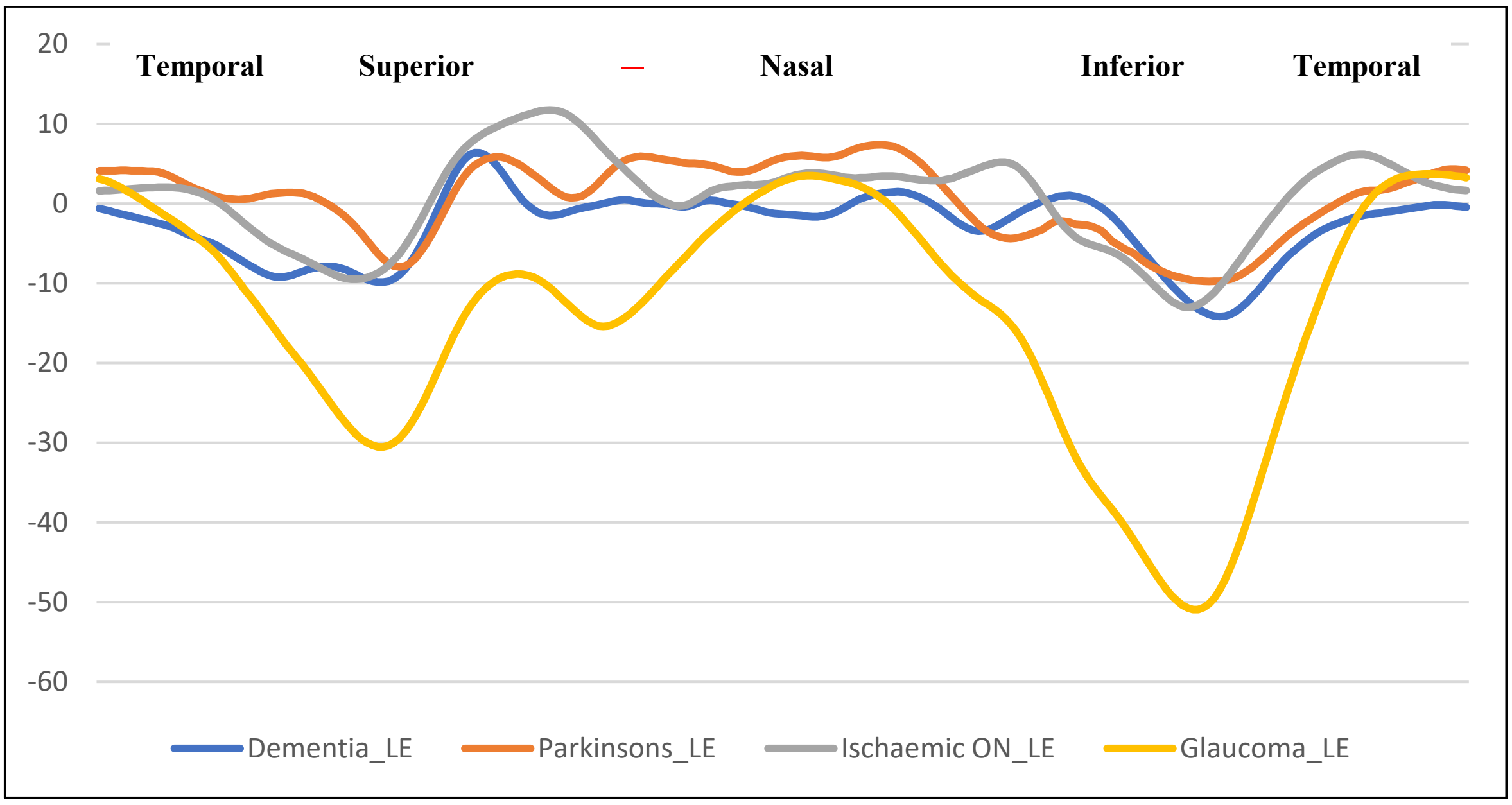

Supplementary Figure 5: *TSNIT plots for retinal neurodegenerative (glaucoma patients) and secondary neurodegenerative conditions (dementia, Parkinson's disease, ION). RE denotes the right eye, while LE refers to the left eye. The dashed region (marked as 'p') indicates the points with a notable distinction (p<0.05) from the normal state.*

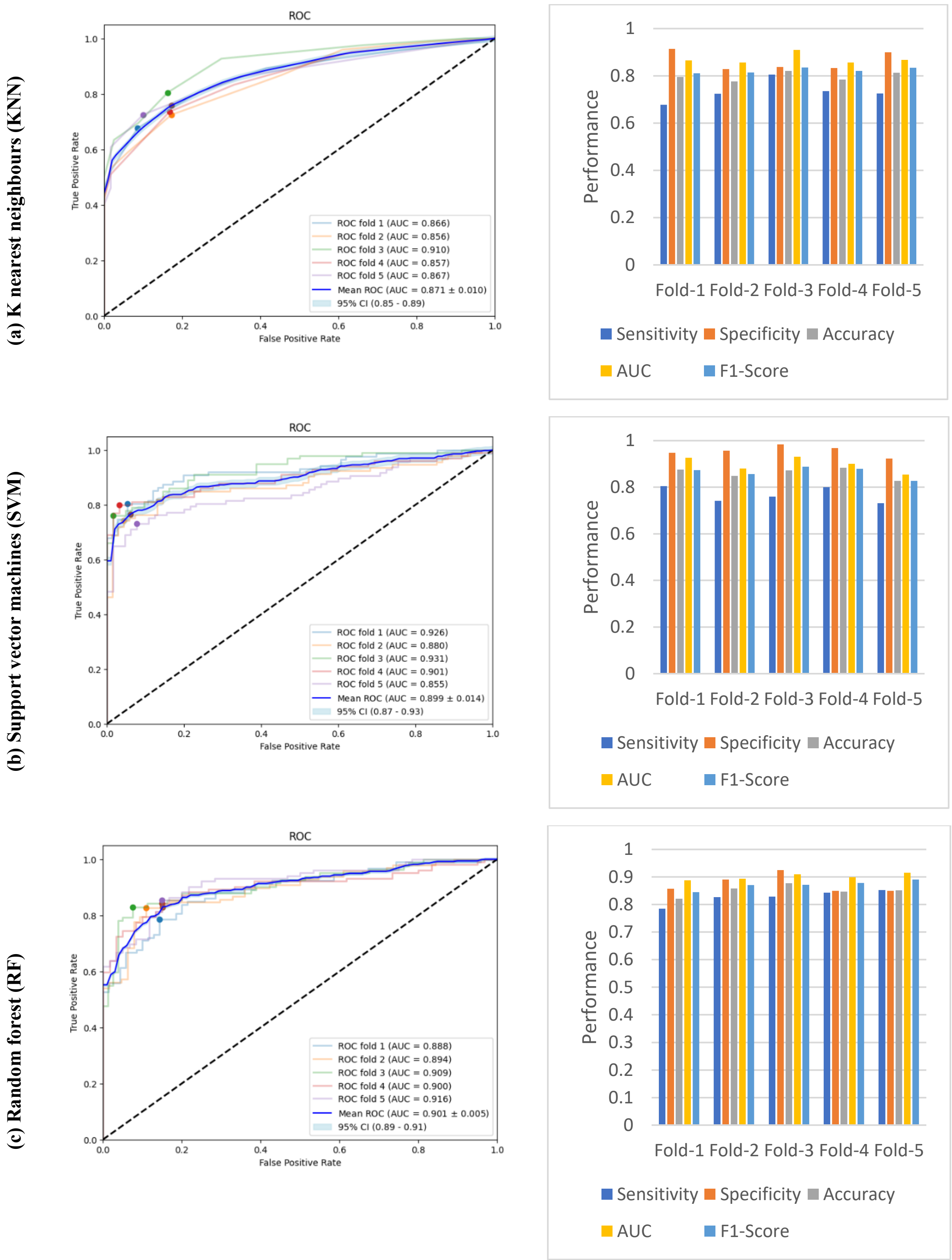

Supplementary Figure 6: *Performance of main classifier models for Optic neuropathy vs Normal (a) K nearest neighbours (KNN) (b) Support vector machines (SVM) (c) Random Forests (RF)*

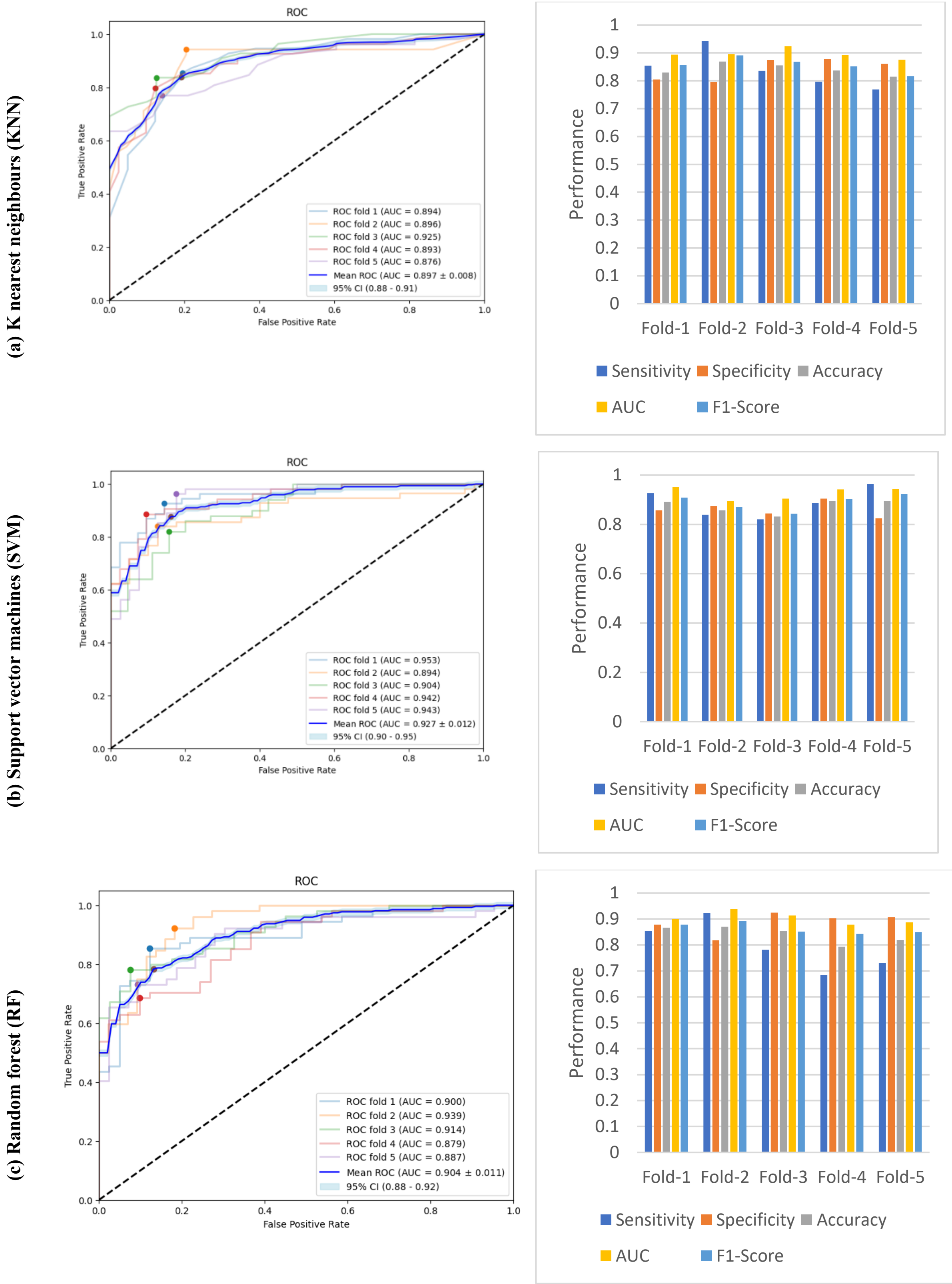

Supplementary Figure 7: *Performance of sub-classifier models for Glaucoma vs Non-glaucoma sub-classification. (a) K nearest neighbours (KNN) (b) Support vector machines (SVM) (c) Random Forests (RF)*

(a) KNN

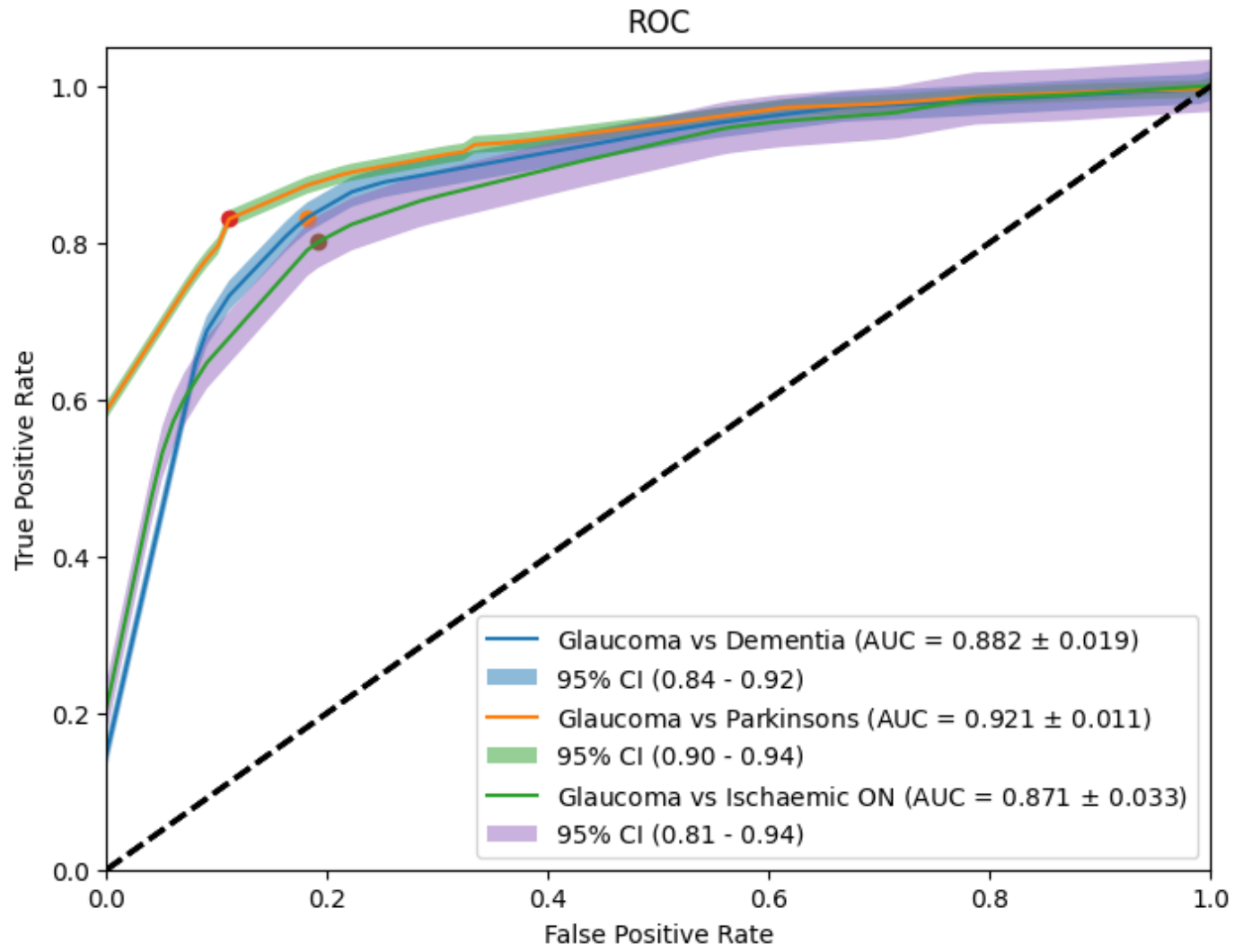

(b) SVM

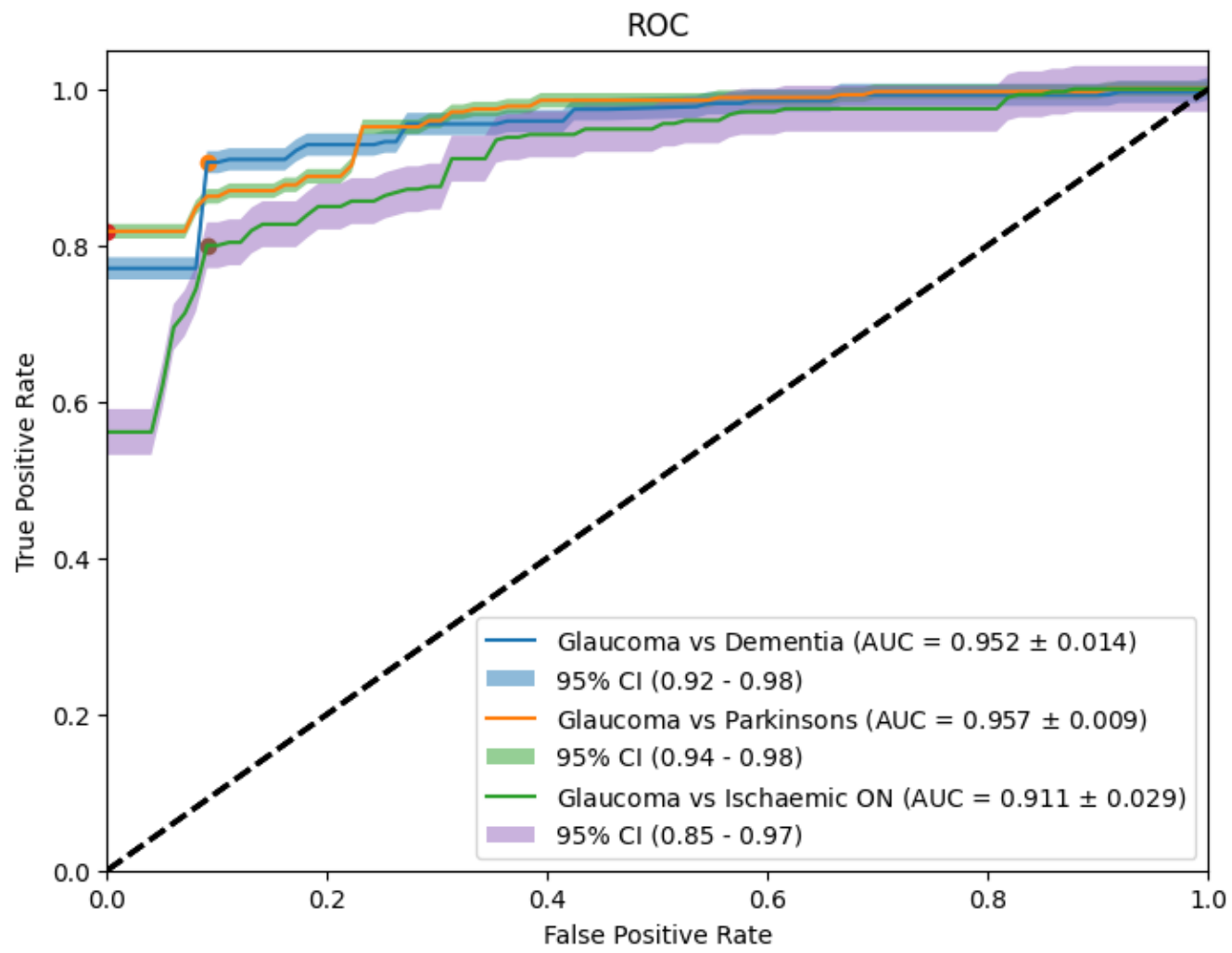

(c) RF

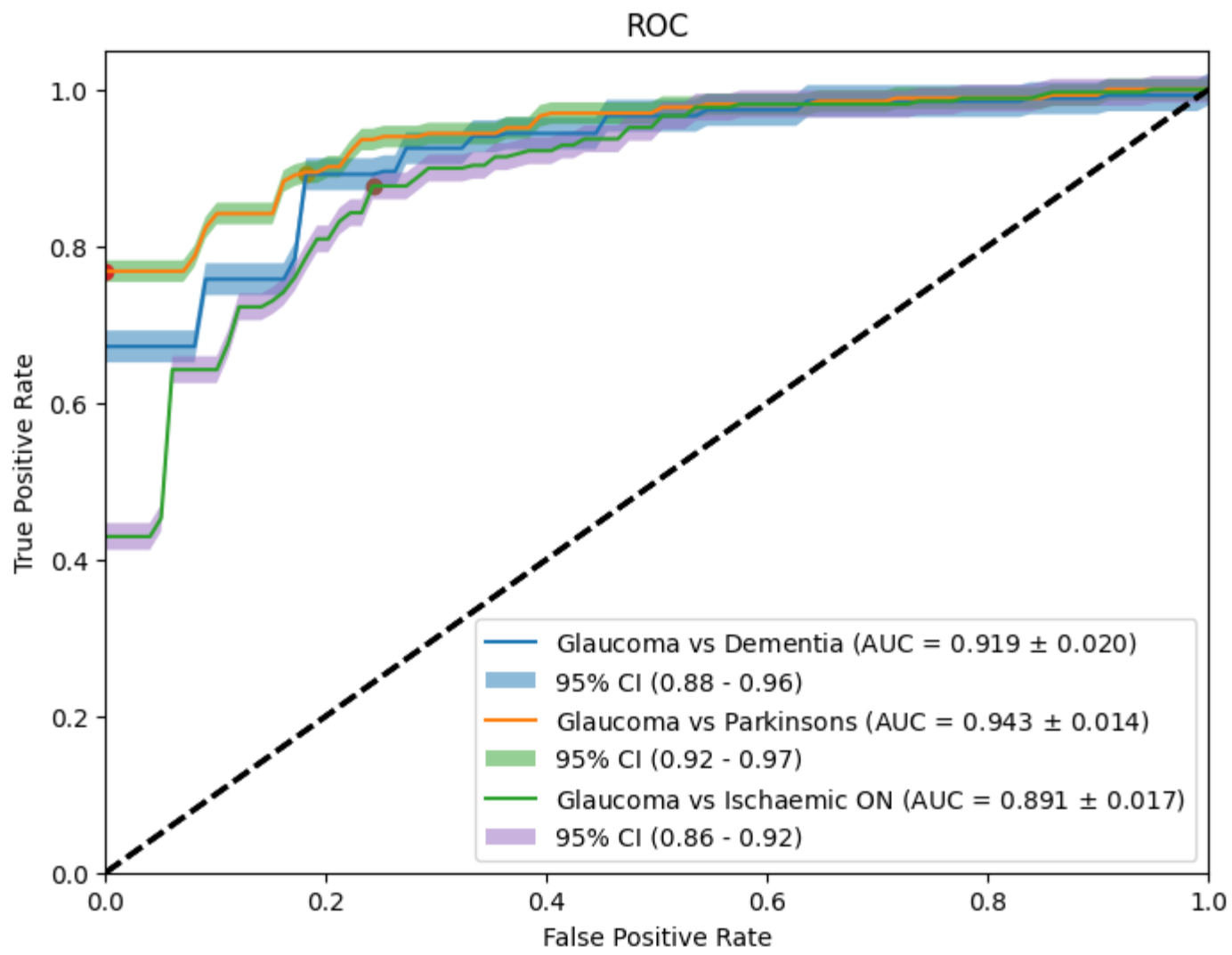

Supplementary Figure 8: *Performance comparison of three machine learning models for glaucoma vs (non-glaucoma) diagnosis- OvO: (a) K nearest neighbours (KNN) (b) Support vector machines (SVM) (c) Random Forests (RF)*

(a) KNN

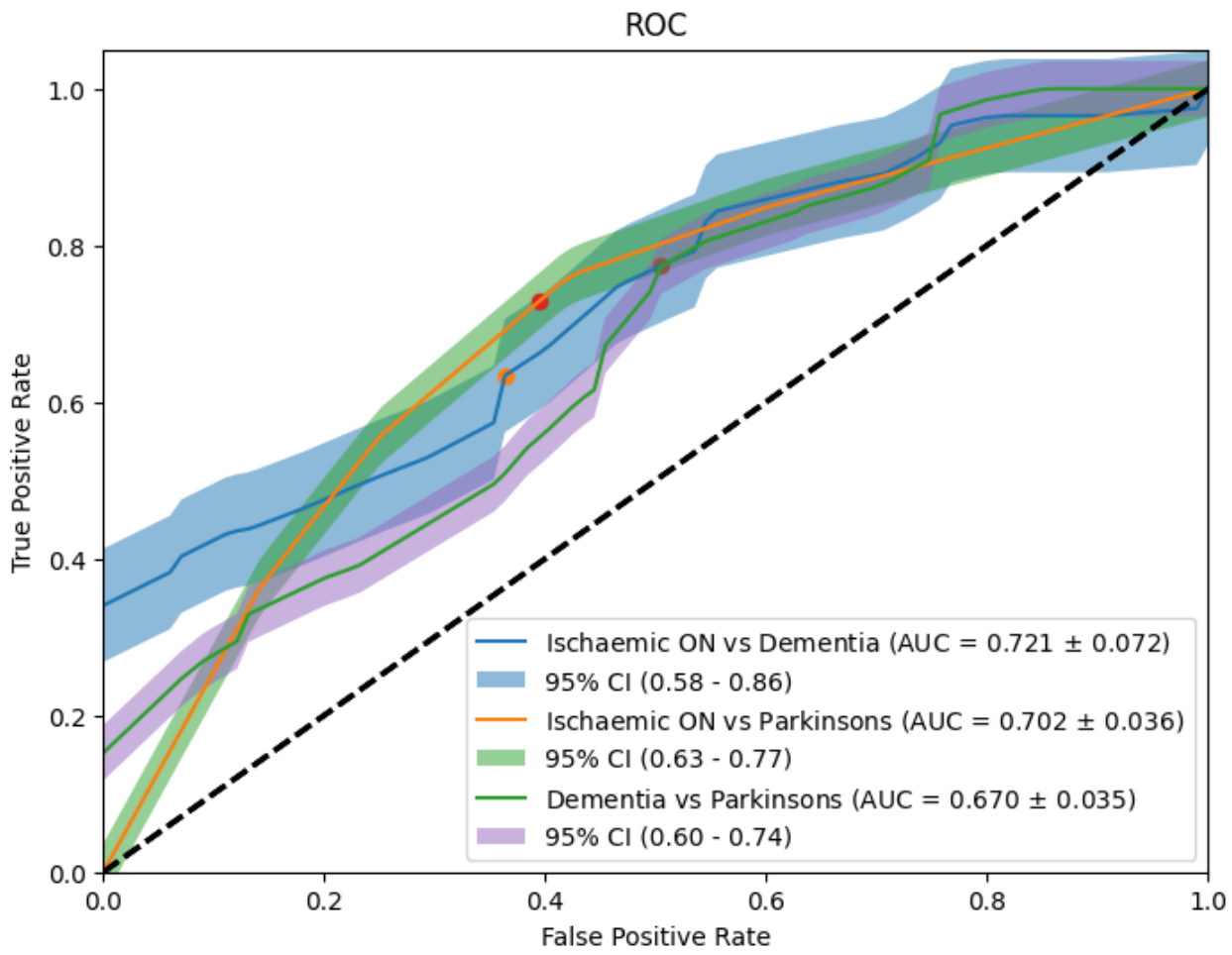

(b) SVM

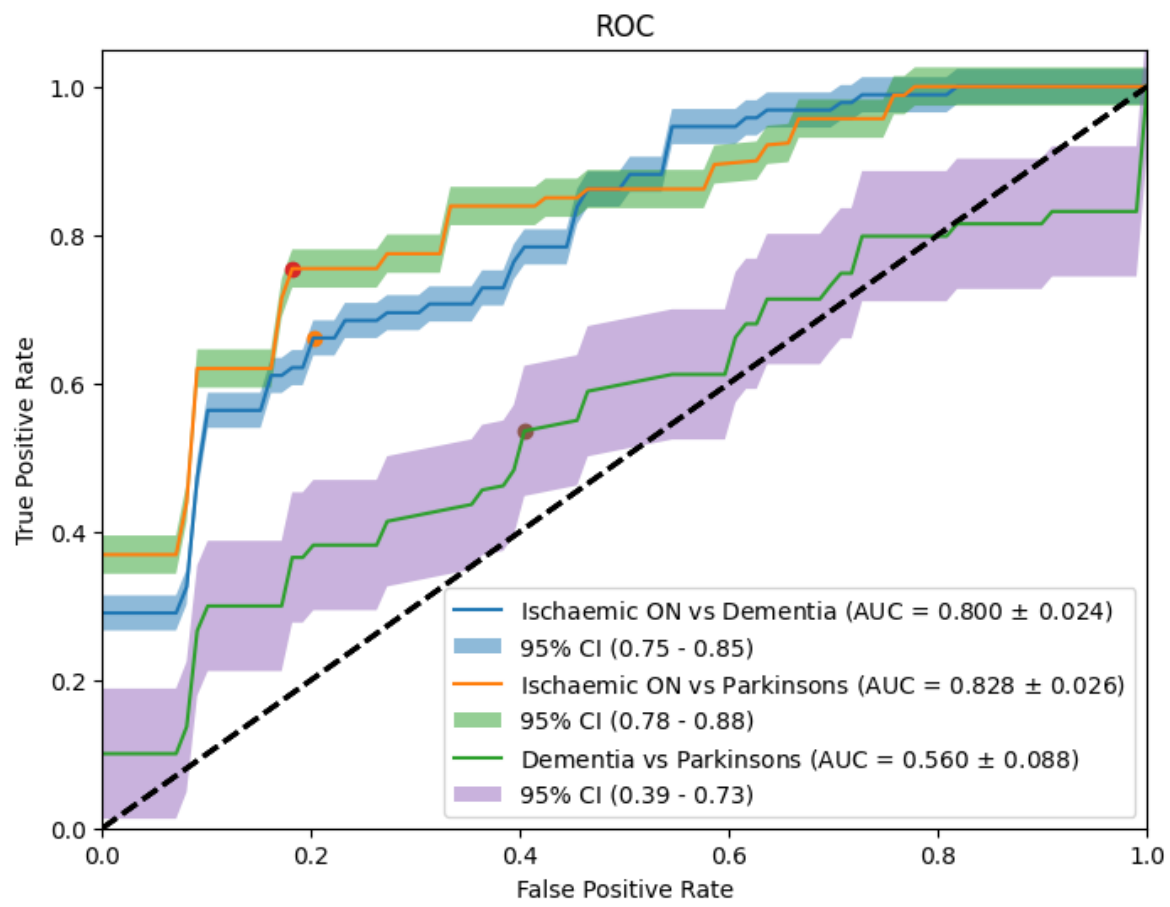

(c) RF

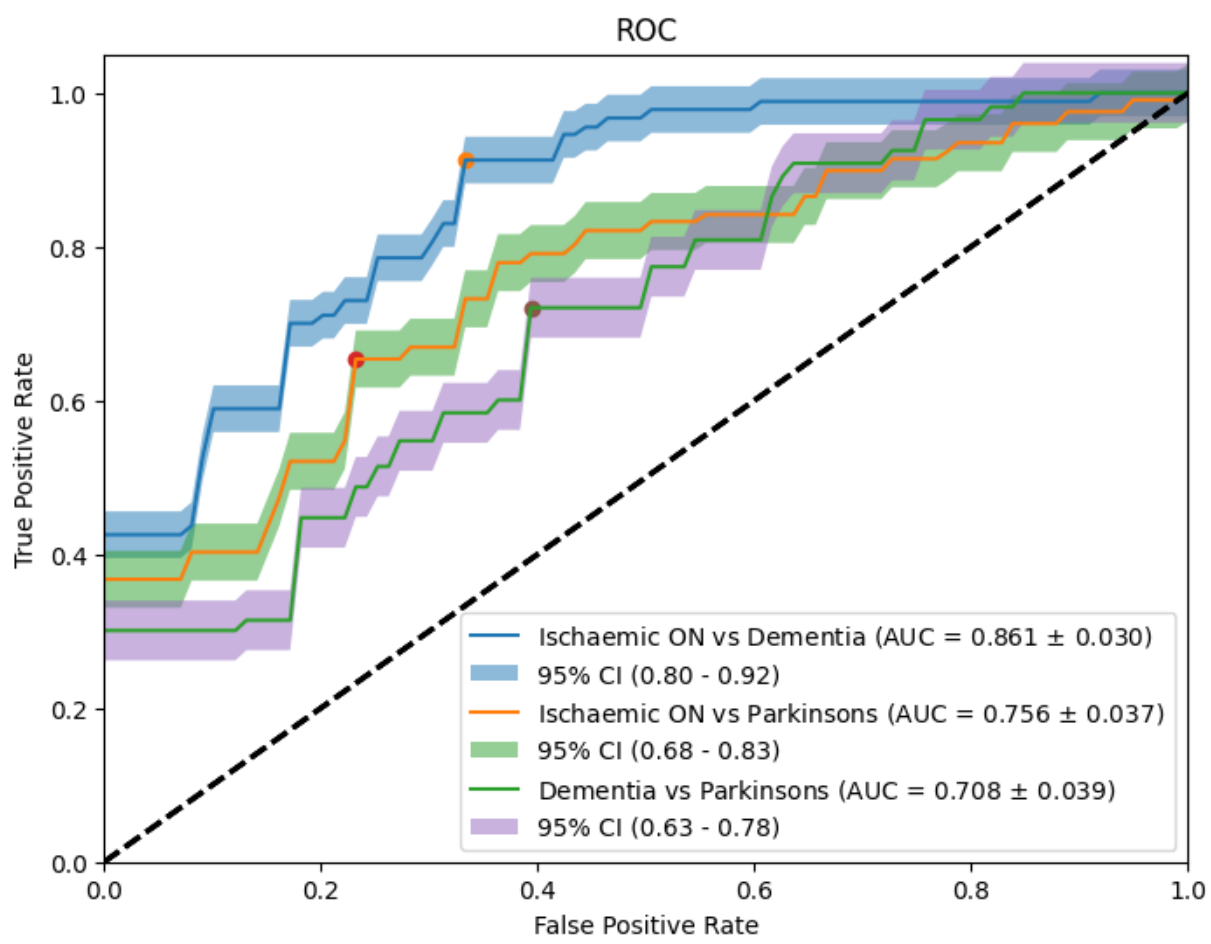

Supplementary Figure 9: *Performance comparison of three machine learning models for Intra-neurodegenerative diseases classification diagnosis- OvO: (a) K nearest neighbours (KNN) (b) Support vector machines (SVM) (c) Random Forests (RF)*

(a) *A sample of a coded patient with masked RNFL provided to the optometrists for grading.*

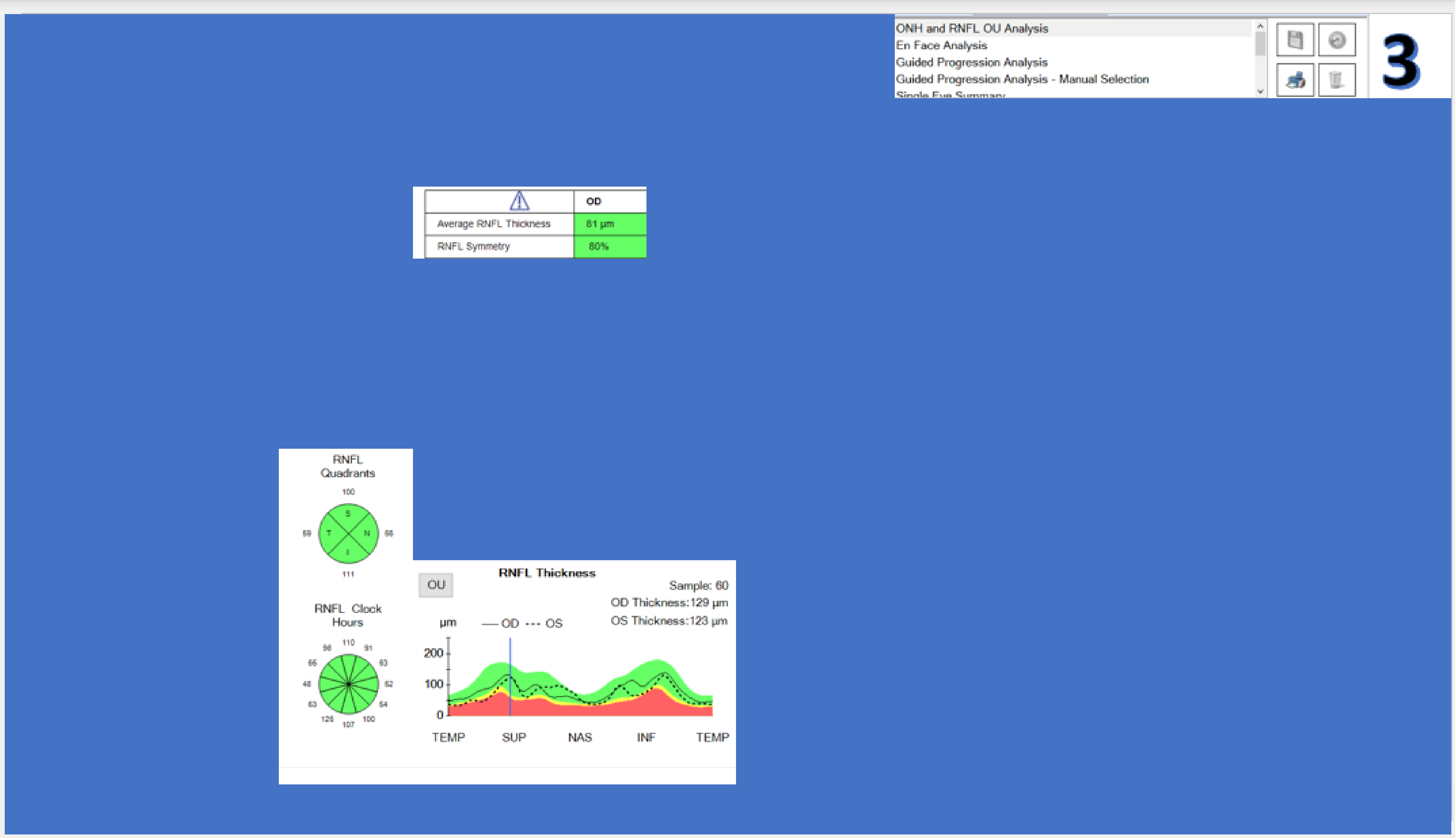

(b) *A sample of unmasked RNFL provided to the optometrists for grading randomly (unmasked-random)*

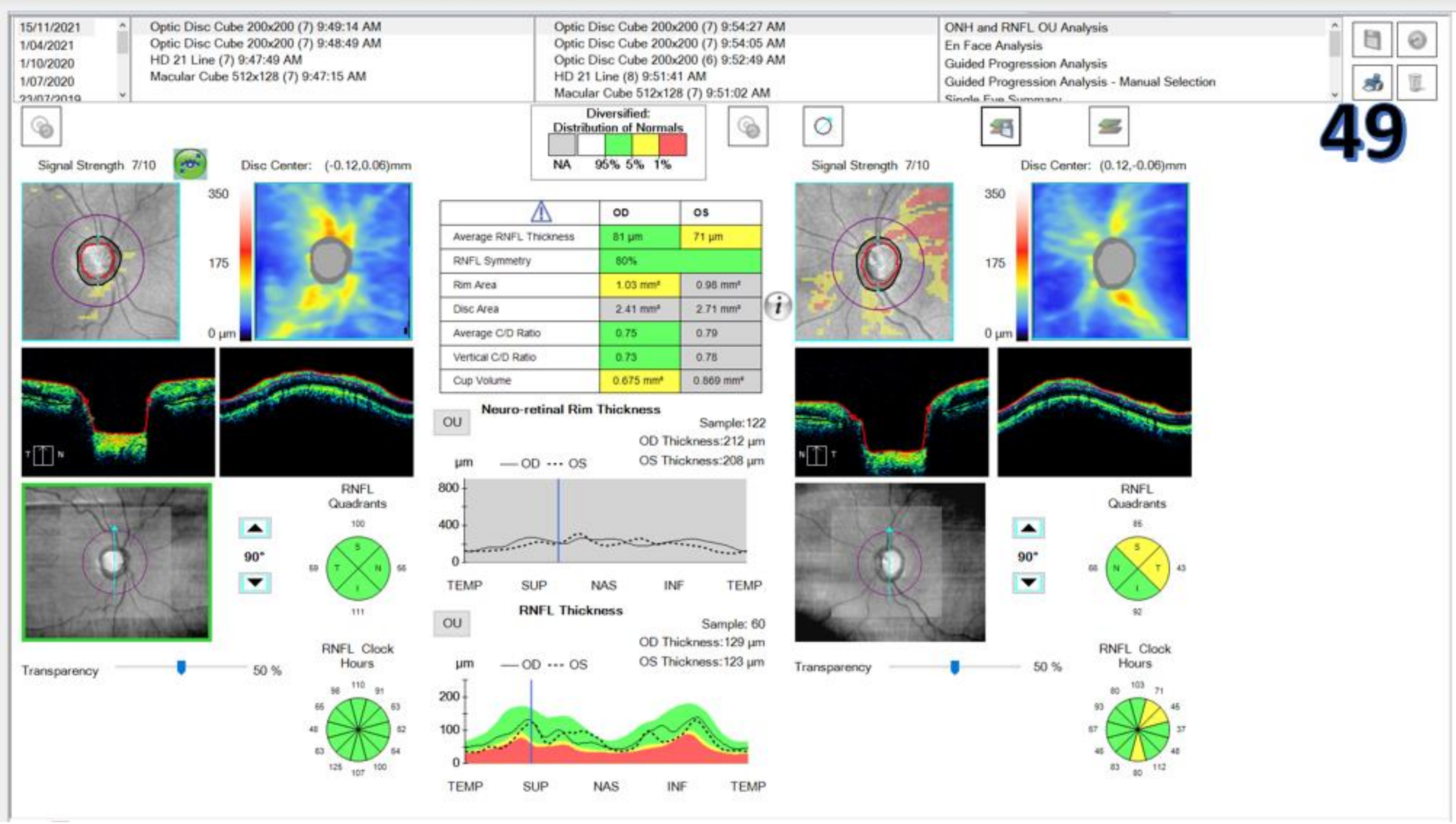

Supplementary Figure 10: *A sample of (a) masked (b) unmasked data provided to the optometrists for grading randomly*
